# Succinate Dehydrogenase Subunit A (SDHA) Mediated Microglia Extracellular Traps Formation Participating in Cerebral Ischemic Reperfusion Injury

**DOI:** 10.1002/advs.202411873

**Published:** 2025-08-20

**Authors:** Lili Zhao, Tao Li, Meijuan Dang, Ye Li, Jialiang Lu, Ziwei Lu, Zhiyang Chen, Qiao Huang, Yujie Chen, Yang Yang, Yuxuan Feng, Xiaoya Wang, Yating Jian, Heying Wang, Yingying Guo, Lei Zhang, Yu Jiang, Songhua Fan, Shengxi Wu, Hong Fan, Fang Kuang, Guilian Zhang

**Affiliations:** ^1^ Department of Neurology the Second Affiliated Hospital of Xi'an Jiaotong University Xi'an Shaanxi 710004 China; ^2^ Department of Neurobiology School of Basic Medicine Fourth Military Medical University Xi'an Shaanxi 710032 China; ^3^ Department of Pediatrics the Second Affiliated Hospital of Xi'an Jiaotong University Xi'an Shaanxi 710004 China

**Keywords:** ischemia reperfusion injury, ischemic stroke, microglia extracellular traps, succinate dehydrogenase

## Abstract

Ischemia reperfusion (I/R) injury associated with recanalization therapy in acute ischemic stroke (AIS) exacerbates the initial brain damage. However, it remains a clinical challenge due to limited understanding of the underlying mechanisms of I/R injury. This study aims to investigate the mechanism of succinate dehydrogenase (SDH)‐mediated succinate oxidation in microglia extracellular traps (MiETs) formation and neuronal injury after cerebra I/R injury. The results show that microglia are the main cell type producing extracellular traps (ETs) at 24 h at cerebral parenchyma after cerebral I/R. Additionally, oxygen glucose deprivation/re‐oxygenation (OGD/R) could induce MiETs formation and increased level of mitochondrial reactive oxygen species (mtROS). Microglia switches toward glycolysis with enhanced SDH activity and upregulated expression of SDH subunit A (SDHA) during MiETosis. Dimethyl malonate (DMM), a competitive SDH inhibitor, could reduce MiETosis by inhibiting succinate oxidation and mtROS production during reperfusion. Furthermore, DMM is found to alleviate neuronal injury after OGD/R exposure and neurological behavior disorders after cerebral I/R, and the effect is similar to MiETosis inhibitor BB‐Cl amidine. These findings reveal a novel functional state of microglia and the role of succinate oxidation in MiETosis after cerebral I/R and provide a novel potential target for the treatment of AIS.

## Graphical abstract

This study investigates the mechanism of succinate dehydrogenase (SDH)‐mediated succinate oxidation in microglia extracellular traps (MiETs) formation and neuronal injury after cerebra I/R injury. Dimethyl malonate (DMM), a competitive SDH inhibitor, could reduce MiETosis by inhibiting succinate oxidation and mtROS production during reperfusion, alleviate neuronal injury and neurological behavior disorders, and the effect is similar to MiETosis inhibitor BB‐Cl amidine.

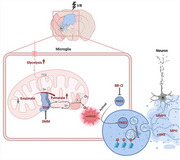

## 1 Introduction

Acute ischemic stroke (AIS) is the leading cause of death and disability worldwide.^[^ ^1^ ^]^ Reperfusion therapy has been recommended by guidelines as one of the most intuitive strategy to reverse ischemic injury by recanalizing occluded blood vessel.^[^ ^2^ ^]^ However, the ischemia‐reperfusion (I/R) injury associated with reperfusion therapy leads to poor prognosis in nearly half of the patients with recanalization.^[^ ^3^ ^]^ Although the mechanisms of I/R injury are multifaceted, neuronal death is the main event relevant to prognosis.^[^ ^4^ ^]^ Although apoptosis, autophagy and programmed necrosis has been deeply studied in cerebral I/R injury, strategies targeted to these forms of cell death achieved limited effects, indicating that uncovering mechanism of the upstream events leading to neuronal death might be a promising alternative.

Microglia, as the principal resident immune cells, is first activated and major immune cell type after AIS.^[^ ^5^ ^]^ After AIS, microglia participate in process of neuronal damage through phenotypic adaptation of polarization,^[^ ^6^ ^]^ autophagy,^[^ ^7^ ^]^ phagocytosis,^[^ ^8^ ^]^ pyroptosis,^[^ ^9^ ^]^ and ferroptosis.^[^ ^10^ ^]^ Polarization emerges as the primary transformation in microglia, yet modulating the polarization phenotype of microglia likewise exert a considerable influence on the progression of central nervous system diseases. The dead and dying microglia could release excessive neurotoxic molecules to damage neurons, aggravate the pathological process, active microglia and forming a vicious cycle. Moreover, microglia in the peri‐infarct area have been previously shown to be critical for functional recovery in animal stroke model.^[^ ^11^ ^]^ Therefore, further studies on death of microglia are of great significance for neuronal survival and neurological recovery.

The production and release of extracellular traps (ETs), termed ETosis, was first described as a suicidal tool to trap and kill bacteria in neutrophils in 2004.^[^ ^12^ ^]^ ETosis is a multistage sequentially developing process, which involves generation of reactive oxygen species (ROS) and/or mitochondria ROS (mtROS), the translocation of the granular enzymes neutrophil elastase (NE) and myeloperoxidase (MPO) to the nucleus, where they promote chromatin decondensation together with peptidyl arginine deiminase (PAD) citrullinating histone.^[^ ^13^ ^]^ ETs are filamentous extracellular web‐like structures of decondensed DNA characterized by the presence of citrullinated histone H3 (citH3), and cytoplasmic proteins, such as NE, cathepsin G, MPO and matrix metalloproteinase (MMP9).^[^ ^13^ ^]^

To date, many cell types have been proved undergoing ETosis in specific pathological environment.^[^ ^14^ ^]^ Microglia was also reported to undergo MiETosis and formed MiETs within stimulation of listeria, IFN‐γ, and dopamine, which played important roles in glioma, meningitis and spinal cord injury.^[^ ^15^, ^16^, ^17^ ^]^ However, it remains unknown whether MiETs or MiETosis participates in cerebral I/R injury and how important it is to neuronal survival in I/R.

In the process of ETs, ROS and/or mtROS plays a central role in steps like cytoskeletal dynamics and cell activation^[^ ^18^, ^19^ ^]^ and contributes to the rupture of nuclear and cell membrane, which facilities DNA mixing with cytosolic protein and releasing from cell.^[^ ^20^, ^21^, ^22^, ^23^ ^]^ Thus, exploring the key factors influencing ROS/mtROS production might provide new insights for understanding and regulating ETosis. It has been demonstrated that succinate oxidation could induce large amount of mtROS by driving reversal electron transport (RET)during reperfusion,^[^ ^24^, ^25^, ^26^ ^]^ and the production of mtROS by RET is thought to be a crucial trigger for the mitochondrial permeability transition pore opening, initiating cell death in I/R injury.^[^ ^11^, ^27^ ^]^ In addition, BV2 cells was reported to experience oxidative phosphorylation (OXPHOS) inhibition and glycolysis strengthening, along with succinate accumulation and ROS generation under hypoxic environment stimulation.^[^ ^28^ ^]^ Therefore, we hypothesize that cerebral I/R could induce accumulation and oxidization of succinate in microglia might mediate MiETs through mtROS production in cerebral I/R injury. We demonstrated that succinate dehydrogenase subunit A (SDHA) could mediate MiETosis through mtROS production and induce neuronal damage after I/R injury *in vivo and in vitro*, indicating that glycosemetabolism regulated MiETosis may be a novel target for the treatment of ischemic stroke.

## 2 Results

### 2.1 MiETs is the Main Source of ETs in Peri‐Infarction Area after Cerebral I/R at 24 h

**Figure 1:**
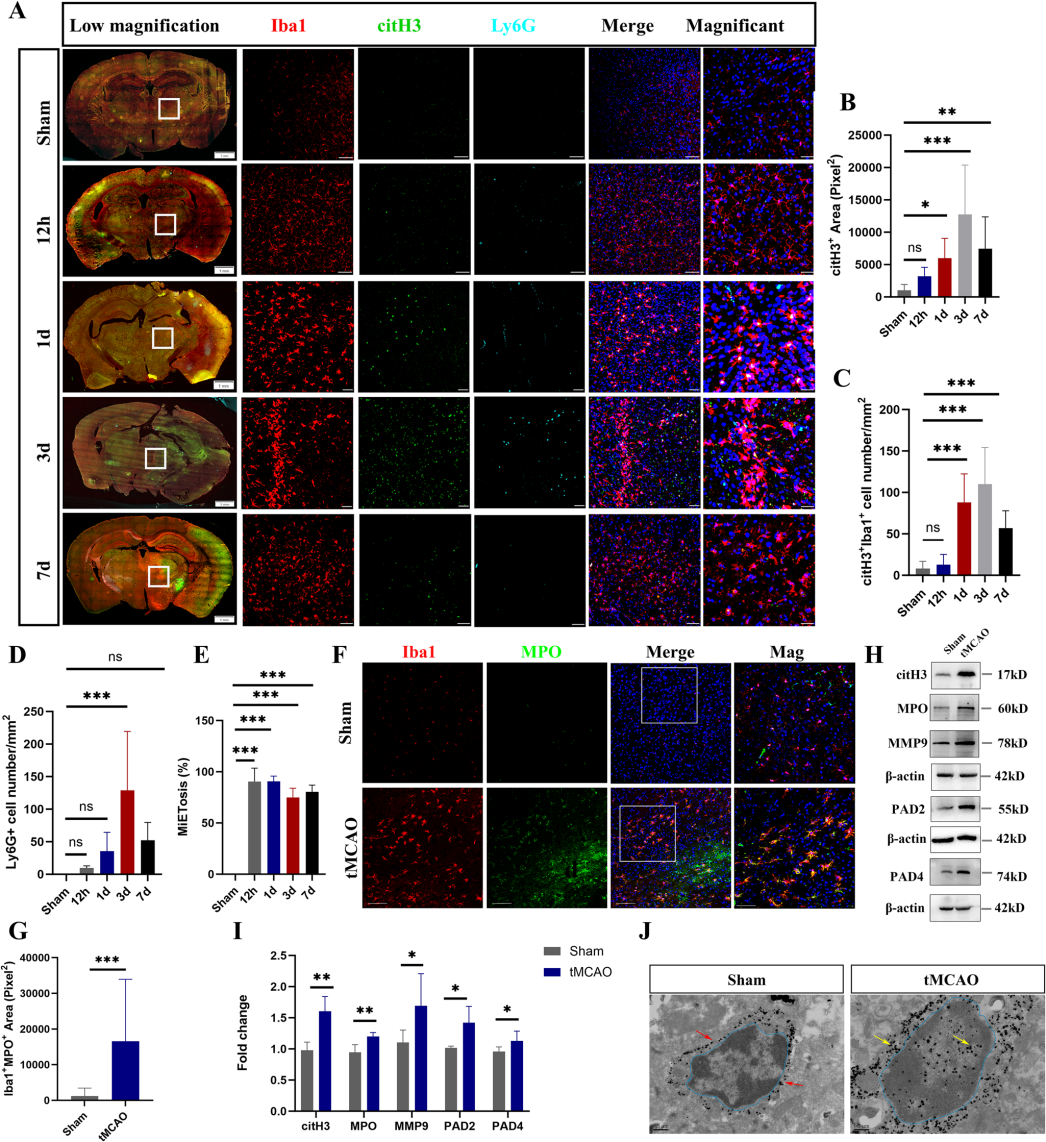
MiETs is the main source of ETs in peri‐infarction area at 24 h after cerebral I/R. A) Immunofluorescence staining for Iba1 (red), Ly6G (cyan) with citH3 (green) at 12 h and 1, 3, 7 days post cerebral I/R in sham and I/R groups in peri‐infarction area. Scale bar = 100 µm. B–E) Quantification of citH3‐positive area, Iba1/citH3 double positive, Ly6G/citH3 double positive and percentage of MiETs (n  =  10–14 images from four animals/group, one‐way ANOVA followed by multiple comparisons). F,G) Representative images of immunostaining of MPO (green) with Iba1 (red), and quantification of MPO/Iba1 double‐positive area at 1 day post cerebral I/R (n  =  10–12 images from four animals/group, one‐way ANOVA followed by multiple comparisons). H) Immune electron microscopy images of microglia at 24 h after cerebral I/R. Cyan line indicate border of nucleus; blue, red and yellow arrows indicate colloidal gold particles of Iba1. Scale bar = 0.5 µm. I) The representative picture of immunoblots for citH3, MPO, MMP9, PAD2, PAD4 and β‐actin at 24 h post cerebral I/R. J) Quantification analysis for the immunoblots in (I) (n  =  4–6/group, *t*‐test) All data are presented as mean ± SD, *p < 0.05, **p < 0.01, ***p < 0.001.

We first detected citH3 expression by performing immunofluorescence (IF) to observe the occurrence of MiETosis after cerebral I/R in mice. Considering neutrophil was an important cell type forming ETs in AIS, we use triple staining of Iba1, Ly6G and citH3 to determine the contribution of microglia and neutrophil to ETosis. The result demonstrated that activated microglia formed a band between normal and ischemic tissue, and citH3 was expressed in microglia from 12 h to 7 days after cerebral I/R (**Figure** **1A**). Quantitative analysis revealed that citH3 positive area, citH3/Iba1‐positive cells and citH3/Ly6G‐positive cells were increased from 12 h, peaked at 3 days and decreased at 7 days after cerebral I/R (Figure 1B–D). Only very limited number of neutrophils was observed in peri‐infarction area at 24 h (Figure 1D), and about 80% of all citH3‐labeled cells in the region were Iba1‐positive from 12 h to 7 days post injury (Figure 1E). We also assessed the expression of citH3 in astrocytes (GFAP^+^) and macrophages/monocytes (LY6C/G^+^) at various time points, indicating 4.72%, 3.18% GFAP/citH3 or Ly6C/G/citH3 double positive cells at 24 h after cerebral I/R (Figure S1, Supporting Information). Considering the very limited number of ETs from neutrophils, astrocytes and macrophages/monocytes at 24 h after cerebral I/R, we choose 24 h as the time point for examination of MiETosis in subsequent experiments. Given the pivotal role of neutrophils in ET formation, we assessed the content of MiETs after antibody‐mediated neutrophil depletion. Notably, neutrophil depletion reduced citH3 but not MiET levels, suggesting a neutrophil‐independent mechanism for MiETs generation (Figure S2, Supporting Information). We also detected the expression of MPO, another marker of MiETosis, in microglia, and the results indicated that the expression of MPO was significantly increased in microglia at 24 h after cerebral I/R (Figure 1F,G). To further confirm the occurrence of MiETosis after cerebral I/R, we observed the ultrastructural changes of microglia. The results revealed decreased chromatin concentration in Iba1‐labled microglia after cerebral I/R (Figure 1H), which are important characteristic of MiETosis. In addition, levels of citH3, MPO, MMP9, PAD2 and PAD4, were all markedly increased in tMCAO group (Figure 1I,J). All together, these results suggested that MiETosis occurred in peri‐infarction area after cerebral I/R injury.

### 2.2 OGD/R Induce MiETosis

**Figure 2:**
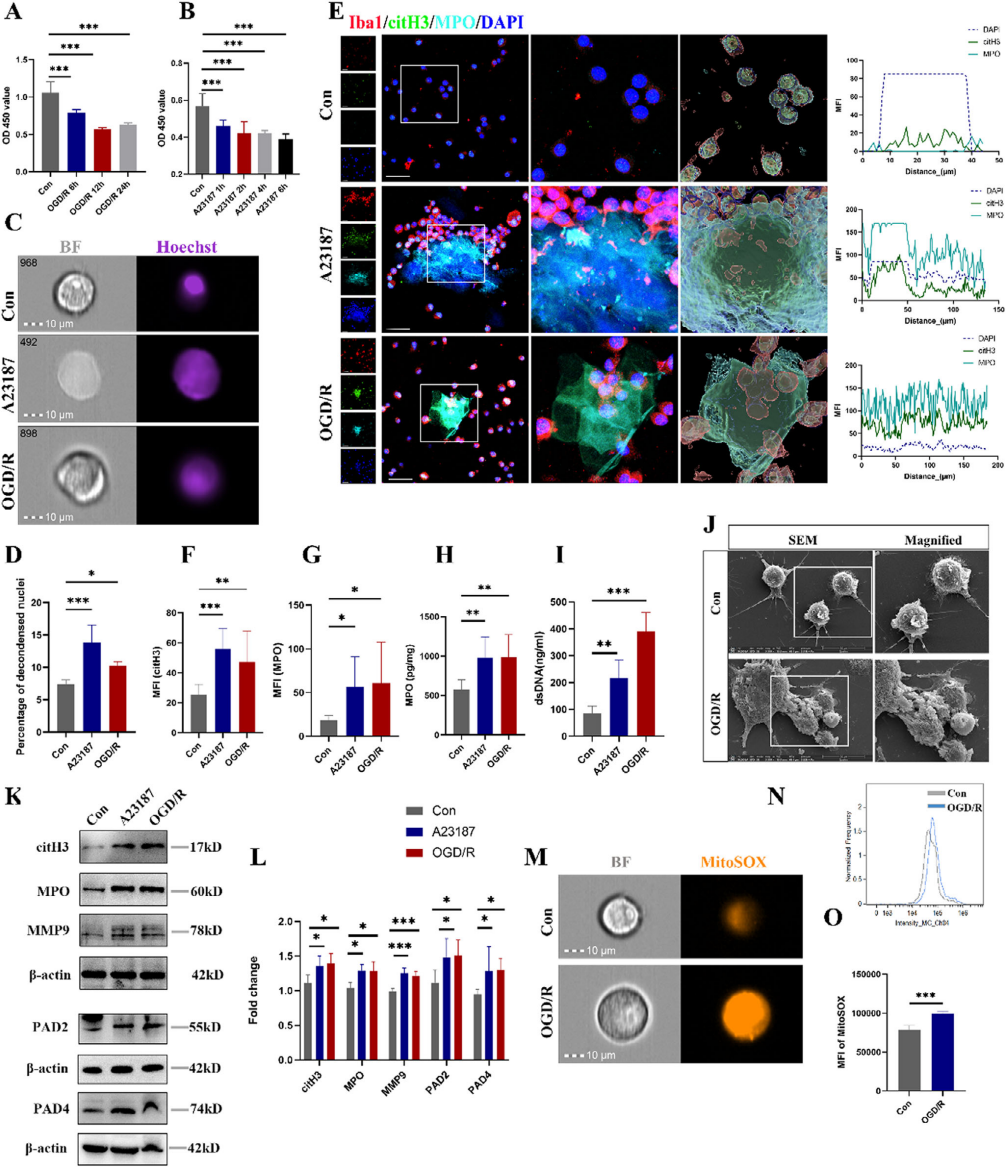
OGD/R induced MiETosis in vitro. A) Cell viability of micoglia was detected by CCK‐8 assay after OGD (4 h) following re‐oxygenation for 6, 12, 24 h, and B) incubation with A23187 (5 µM) for 1, 2, 4, 6 h. C) Representative images of microglia following incubation with the Hoechst 33342 show the presence of nuclear decondensation at 40 × magnification. Scale bar = 10 µm. D) Quantification of percentage of microglia undergoing nuclear decondensation. E) Representative images of Iba1, citH3 and MPO triple‐stained microglia, and colocalization analysis of MPO, citH3 and DNA in three group. Scale bar = 50 µm F,G) Quantitative analysis of MPO and citH3 mean immunofluorescence intensities (MFI) in each group of microglia. H) MPO, I) ds‐DNA) concentrations in microglia culture supernatant, as detected by ELISA. J) Scanning electron microscopy (SEM) results demonstrated that microglia released porous, protein‐rich extracellular structures following OGD/R treatment. K,L) Representative immunoblot and statistical analysis of citH3, MPO, MMP9, PAD2, PAD4 and β‐actin after OGD/R exposure. M) Representative images of microglia following incubation with the fluorescent probe mitoSOX show the production of mtROS at 40 × magnification. Scale bar = 10 µm. N) Single‐color histogram of mitoSOX fluorescence intensity. All images are representative and were chosen at random. O) Quantification of mean intensities of mitoSOX. All experiments were performed at least three independent times. All data are presented as mean ± SD, *p < 0.05, **p < 0.01, ***p < 0.001.

We further investigated whether OGD/R could induce MiETosis to explore the mechanism of MiETosis. First, we treated primary microglia with the varying reperfusion time, and different concentrations of ETosis inducer A23187 which was applied as a positive control. As shown in **Figure** **2**A,B, we found that cell viability was decreased by ≈50% at 12 h after reperfusion and 4 h after A23187 treatment. Subsequently, these two conditions were selected for subsequent experiment. Changes in nuclear decondensation were assessed with imaging flow cytometry (IFC). A change in nuclear morphology from well‐organized to swelled and fuzzy‐looking was regarded as an early marker of neutrophil undergoing NETosis.^[^ ^29^ ^]^ This change can be monitored by using “Bright Detail Intensity_R3” (BDI_R3) feature in the IDEAS software that identifies normal nuclei as high or variable BDI and low area, and fuzzy, decondensed nuclei as wide areas of low BDI. The result showed that A23187 and OGD/R induced significant increase in the percentage of microglia undergoing nuclear decondensation (Figure 2C,D). Using IF microscopy, we observed MiETs—web‐like DNA structures co‐localizing with citH3 and MPO. Besides, IF analysis showed that A23187 and OGD/R treatment could significantly induce the expression of citH3 and MPO in microglia (Figure 2E–G). Meanwhile, the levels of ds‐DNA and MPO were significantly elevated in supernatant of A23187 and OGD/R‐treated microglia (Figure 2H,I). SEM imaging revealed that microglia actively extruded porous, protein‐laden structures upon OGD/R challenge (Figure 2J). Protein levels of citH3, MPO, MMP9, PAD2 and PAD4 were significantly increased after A23187 and OGD/R exposure (Figure 2K,L). Moreover, there were significant increase in the level of cytosolic ROS (Figure S3, Supporting Information) and mtROS (Figure 2M–O) after OGD/R treatment. The above results indicate a role of MiETosis in I/R injury.

### 2.3 Microglia Switch Toward Glycolysis Following I/R

**Figure 3:**
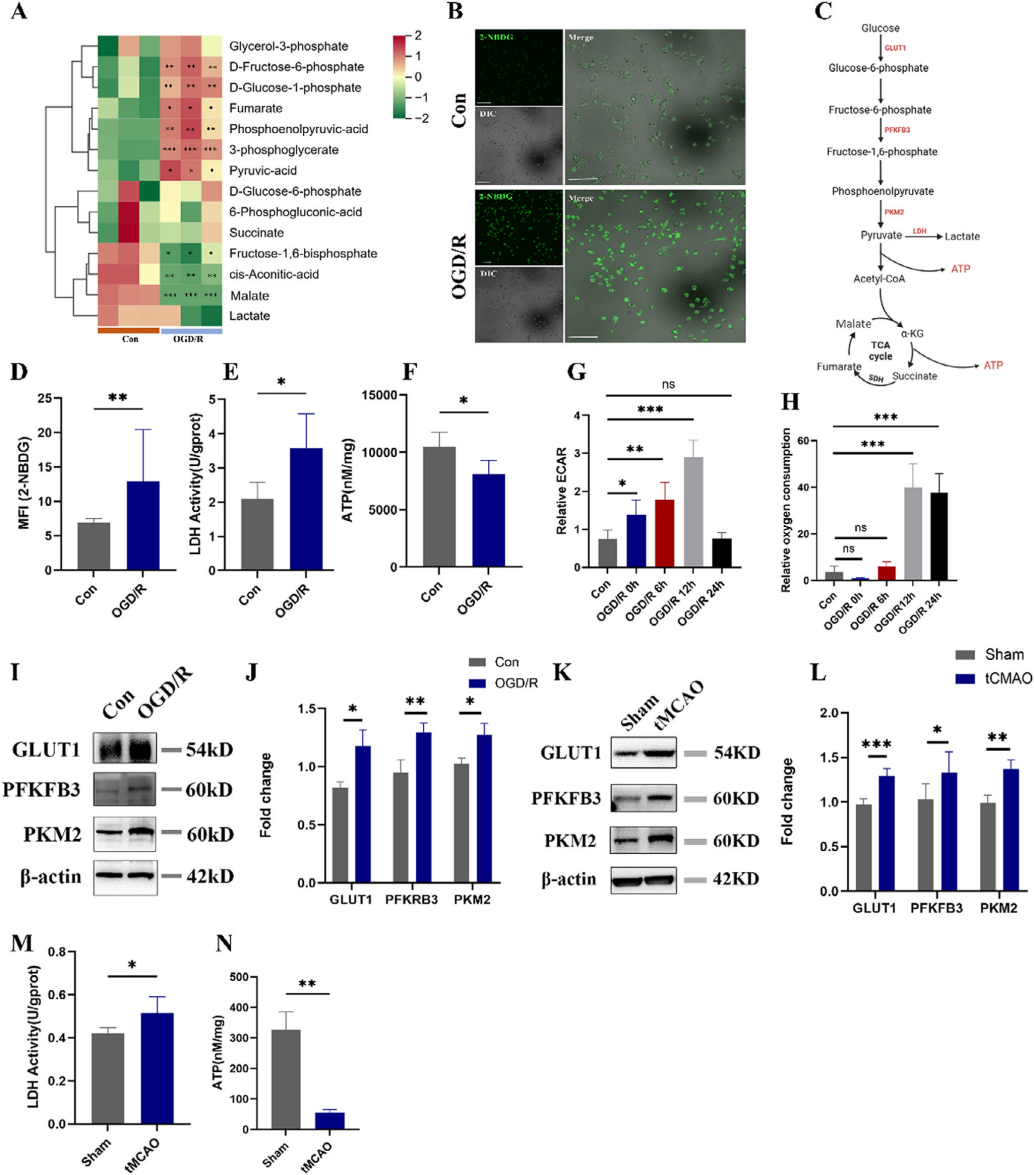
Microglia switched toward glycolysis after OGD/R and cerebral I/R. A) OGD/R induces glycolytic activation and TCA cycle suppression in microglia (targeted metabolomics). B) Levels of glucose uptake in microglia was stained with 2‐NBDG. D) Quantification of MFI of 2‐NBDG between control, and OGD/R groups. E) LDH and F) ATP levels in microglia were detected by ELISA between control, and OGD/R groups. C) Schematic diagram of enhanced glycolysis in microglia. G) Microglial metabolic dynamics post‐reperfusion (ECAR). H) Microglial metabolic dynamics post‐reperfusion (OCR). I,J) The representative immunoblots and statistical analysis of GLUT1, PFKFB3, PKM2 and β‐actin in microglia after OGD/R treatment. All experiments were performed at least three independent times. K,L) The representative immunoblots and statistical analysis of GLUT1, PFKFB3, PKM2 and β‐actin in tissues from peri‐infarction region after cerebral I/R for 24 h (n  =  4–6/group, t test). M,N) The content of LDH and ATP in peri‐ischemic core area tissues between Sham and tMCAO group (n  =  6/group, t test). All data are presented as mean ± SD, *p < 0.05, **p < 0.01, ***p < 0.001.

Previous evidences showed that inhibition of glycolysis by 2‐Deoxy‐D‐Glucose (inhibitor of hexokinase2), glucose‐6‐phosphate dehydrogenase inhibitor,^[^ ^30^ ^]^ PFK15 (inhibitor of fructose‐2,6‐biphosphatase 3, PFKFB3)^[^ ^31^ ^]^ could significantly suppress ETs formation, indicating that glucose metabolism and its related ROS production was vital for ETosis. Thus, we supposed that metabolic switch in microglia might involve in MiETosis. To further elucidate the potential role of glucose metabolism in MiETosis, we first tried to figure out whether I/R induced similar metabolic switch in microglia after OGD/R treatment. The reprogramming of microglial glucose metabolism post‐OGD/R was evidenced by targeted metabolomics. The results showed that significant increases in glucose‐6‐phosphate, fructose‐6‐phosphate, phosphoenolpyruvic‐acid, pyruvate, 3‐phosphyoglycerate et al. were observed in OGD/R‐treated microglia compared to controls, confirming enhanced glycolytic flux. While levels of malate, and succinate remained unchanged, indicating no compensatory activation of oxidative phosphorylation (**Figure** **3**A). Also, the results indicated increased levels of glucose uptake shown by 2‐NBDG staining and lactate‐dehydrogenase (LDH) activity, and decreased adenosine‐triphosphate (ATP) production in OGD/R treated microglia (Figure 3B–F). Longitudinal metabolic profiling showed time‐dependent alterations in microglial bioenergetics: progressive elevation of extracellular acidification rate (ECAR) during the first 12 h of reperfusion (peaking at 12 h) followed by normalization at 24 h, while oxygen consumption rate (OCR) displayed delayed augmentation becoming significant at 12–24 h post‐reperfusion (Figure 3G,H). Additionally, protein levels of glucose transporter type 1 (GLUT1), PFKFB3 and pyruvate kinase isozyme typeM2 (PKM2), key enzymes of glycolysis, were also markedly increased (Figure 3I,J). Similarly, the expression of GLUT1, PFKFB3, and PKM2 were significantly higher in tMCAO mice than that in sham group (Figure 3K,L). Meanwhile, elevated LDH activity and decreased ATP level were detected in tMCAO mice (Figure 3M,N). These results indicated that reprogramming of glucose metabolism with enhanced glycolysis in microglia following I/R. Moreover, the suppression of MiETs markers by 2‐DG (a glycolysis inhibitor) indicates that OGD/R‐triggered metabolic shifts in microglia is critically involved in MiETosis (Figure S4, Supporting Information).

### 2.4 Succinate Dehydrogenase Mediated the Oxidation of Succinate in Microglia at Reperfusion

**Figure 4:**
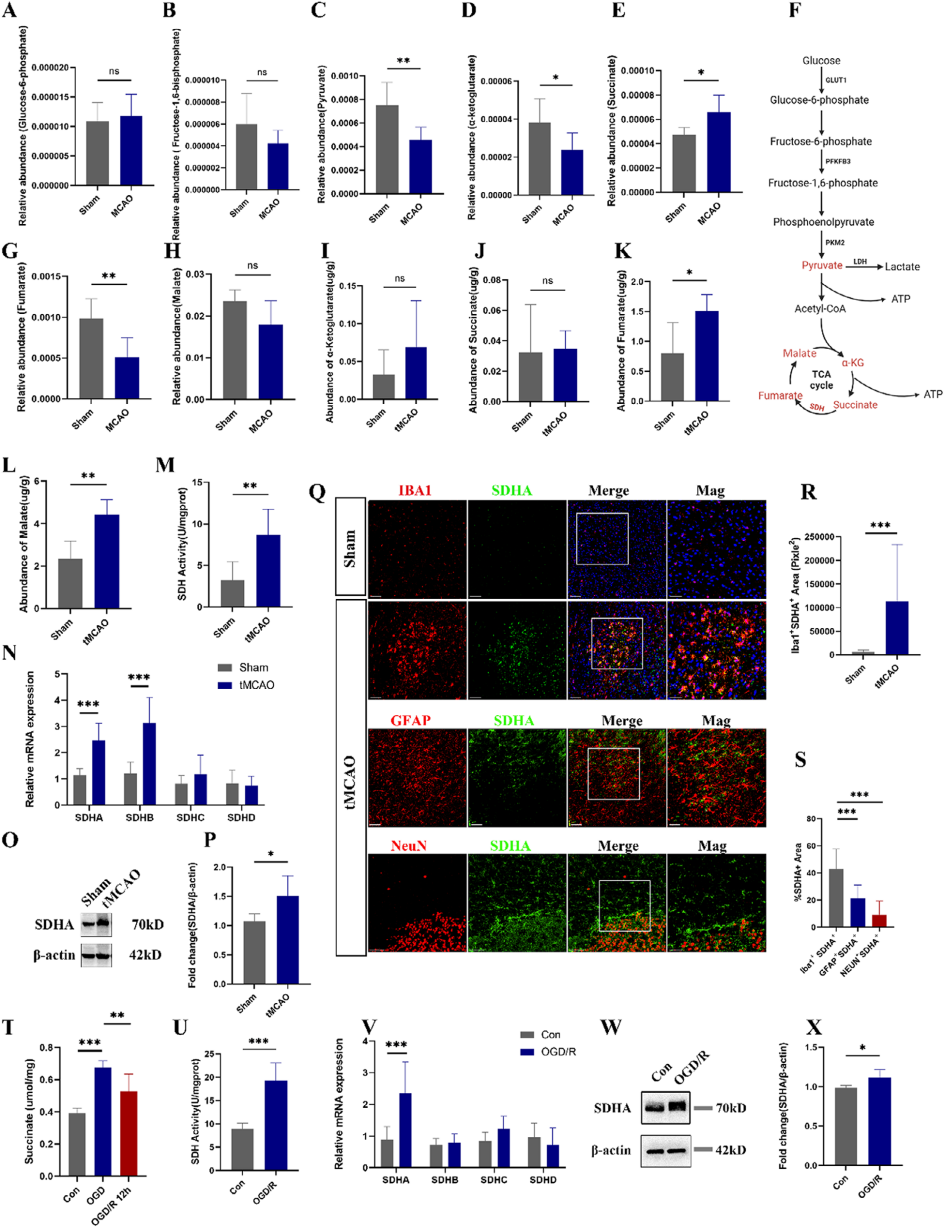
Succinate dehydrogenase mediated the oxidation of succinate in microglia at reperfusion. The relative abundance of A) glucose‐6‐phosphate, B) Fructose‐1,6‐bisphosphate, and C) pyruvate, D) α‐ketoglutarate (α‐KG), E) succinate, G) fumarate, H) malate in peri‐infarction area in Sham and MCAO mice. F) Schematic diagram of changes in substrate levels of glucose metabolism (n  =  6/group, one‐way ANOVA or t test). The content of I) α‐ketoglutarate (α‐KG), J) succinate, K) fumarate, L) malate after reperfusion (n  =  6/group, one‐way ANOVA or *t* test). M) SDH activity in Sham and I/R group at 24 h after reperfusion (n  =  6/group, t test). (N) mRNA expression of SDH subunits genes SDHA, SDHB, SDHC, SDHD at 24 h post cerebral I/R in sham and I/R groups (n  =  5–8/group, one‐way ANOVA or *t* test). O,P) The representative immunoblots and statistical analysis of SDHA and β‐actin in tissues from peri‐infarction region after cerebral I/R for 24 h (n  =  5–7/group, t test). Q–S) Representative images of immunostaining of SDHA (green) with Iba1, GFAP, NEUN, and quantification of SDHA/Iba1 double‐positive area, and percentage of SDHA/Iba1 double‐positive area, SDHA/GFAP double positive area, SDHA/NEUN double positive area at 1 day post cerebral I/R (n  =  12–14 images from four animals/group, one‐way ANOVA followed by multiple comparisons). Scale bar = 100 µm. T) The content of succinate at OGD and reperfusion. U) SDH activity in Con and OGD/R group (t test). V) mRNA expression of SDH subunits genes SDHA, SDHB, SDHC, SDHD after OGD/R exposure (one‐way ANOVA or t test). W,X) The representative immunoblots and statistical analysis of SDHA and β‐actin in microglia after OGD/R treatment. All experiments were performed at least three independent times. All data are presented as mean ± SD, *p < 0.05, **p < 0.01, ***p < 0.001.

We next performed LC‐MS to figure out the key metabolites in ischemia and reperfusion. As shown in **Figure** **4**A,B,H, the relative abundance of glucose‐6‐phosphate, fructose‐1,6‐bisphosphate and malate had no significant change in MCAO group compared to sham group, while pyruvate, α‐ketoglutarate (α‐KG), and fumarate in peri‐infarction tissues decreased after ischemia (Figure 4C,D,G). Under this condition of glycometabolic substrate depletion, only succinate accumulated in ischemia brain tissues (Figure 4E) which is considered as a universal metabolic signature of ischemia.^[^ ^24^ ^]^ However, the content of succinate had no significant difference between tMCAO group and sham group after reperfusion (Figure 4J) companied with elevated level of fumarate, malate in tMCAO group (Figure 4K,L), which indicated that accumulated succinate oxidized by succinate dehydrogenase (SDH) during reperfusion. Besides, there was no significant difference in the content of α‐KG in both groups (Figure 4I).

We then examined the activity and expression of SDH in brain tissue and cultured microglia. The results showed that SDH activity was higher in tMCAO group than that in sham group (Figure 4M). The mRNA levels of SDHA and SDHB were upregulated after cerebral I/R (Figure 4N). Given SDHA was the largest subunit of SDH and contained the binding site of succinate, we selected SDHA as the primary target for further investigation. The result of WB analysis revealed an elevated expression level of SDHA protein in the peri‐infarction tissue subsequently to cerebral I/R (Figure 4O,P). The results of double staining showed that the expression of SDHA was mainly in Iba1‐positive cells. Quantitative analysis revealed that area of both SDHA‐ and Iba1‐positive cells were significantly increased in the peri‐infarction area after cerebral I/R (Figure 4Q,R). IF analysis demonstrated that ≈43.10% of SDHA‐positive cells were Iba1‐positive, while 21.49% were GFAP‐positive and 9.29% were NEUN‐positive (Figure 4S). Our in vitro results further indicated succinate accumulating after OGD and decreased after reperfusion (Figure 4T), a potentiation of SDH activity alongside an upregulation of SDHA subunit mRNA and protein expression level in microglia after OGD/R exposure (Figure 4U–X), indicating the potential role of SDHA in MiETosis during the reperfusion.

### 2.5 Inhibition of Succinate Oxidation Suppress MiETosis

SDH mediated succinate oxidation is consider to be the main source of mtROS production during reperfusion,^[^ ^32^ ^]^ and the latter was regarded as a key player in ETosis. Therefore, we supposed that SDH might participated in MiETosis during reperfusion. DMM, a competitive inhibitor of SDH, was applied to explore the effect of succinate oxidation on MiETosis, which could rapidly hydrolyze to malonate and cross the blood‐brain barrier,^[^ ^33^ ^]^ and was reported to ameliorate renal I/R injury when administered at reperfusion.^[^ ^34^ ^]^ Besides, BB‐Cl, an inhibitor of PADs, could significant inhibit ETosis, which was used as a positive control.

**Figure 5:**
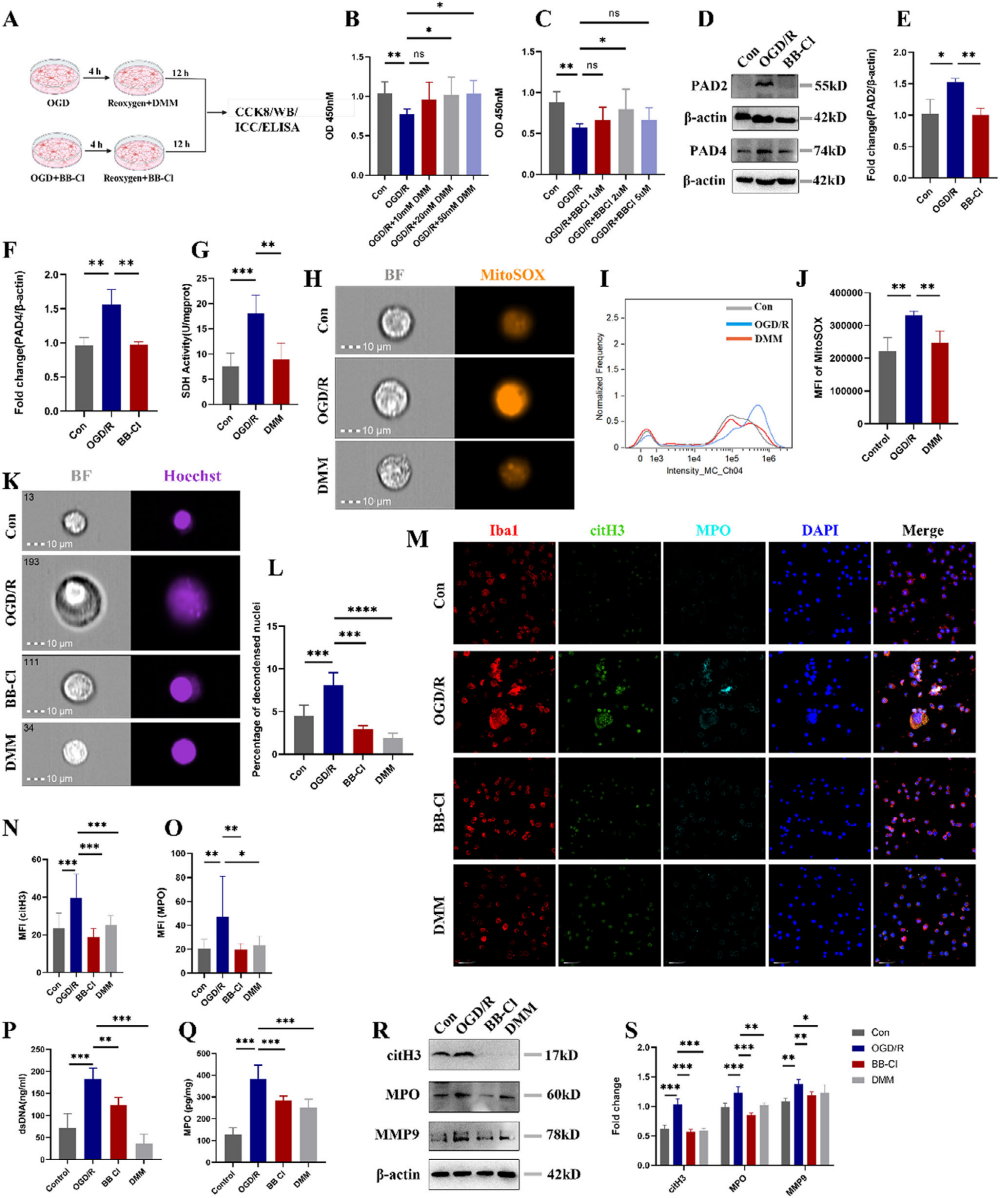
Inhibition of succinate oxidation and PADs mitigated OGD/R induced MiETosis. A) Schematic illustration of the experimental design framework for this section. B) Cell viability of microglia was detected by CCK‐8 assay following incubation with 10, 20, 50 mM DMM at re‐oxygenation. C) Cell viability of microglia was detected by CCK‐8 assay following incubation with 1, 2 and 5 µM BB‐Cl. D,E) The representative immunoblots and statistical analysis of PAD2, PAD4 and β‐actin in microglia after BB‐Cl intervention. F) SDH activity in microglia treated with OGD/R and 20 mM DMM. G) Representative images of imaging flow cytometry (IFC) results of microglia following incubation with the fluorescent probe mitoSOX show the reduction of mtROS after DMM treatment. Scale bar = 10 µm. H) Single‐color histogram of mitoSOX fluorescence intensity in Con, OGD/R, and DMM groups. All images are representative and were chosen at random. I) Quantification of mean intensities of mitoSOX in the three groups. J) Representative images of microglia following incubation with the Hoechst 33342 show the presence of nuclear decondensation at 40 × magnification. Scale bar = 10 µm. K) Quantification of percentage of microglia undergoing nuclear decondensation in Con, OGD/R, DMM and BB‐Cl groups. L) Representative images of Iba1, citH3 and MPO triple‐stained microglia in four group. Scale bar = 50 µm. M, N) Quantitative analysis of MPO and citH3 mean immunofluorescence intensities (MFI) in each group of O) microglia. ds‐DNA, P) MPO concentrations in microglia culture supernatant, as detected by ELISA. Q,R) Representative immunoblot and statistical analysis of citH3, MPO, MMP9 and β‐actin after DMM and BB‐Cl treatment. All experiments were performed at least three independent times. All data are presented as mean ± SD, *p < 0.05, **p < 0.01, ***p < 0.001.

As shown in **Figure** **5**A, DMM was added into the culture media at reoxygenation while BB‐Cl was always in the system. First, we found that 20 mM DMM and 2 uM BB‐Cl significantly alleviated the decline of microglia activity induced by OGD/R (Figure 5B,C). Then, we chose 20  mM DMM and 2 uM BB‐Cl for subsequent experiments. The result showed that 2 uM BB‐Cl could markedly decrease OGD/R induced PAD2 and PAD4 expression (Figure 5D,E). ELISA and IFC analysis indicated that 20 mM DMM could significant decrease SDH activity and mtROS level induced by OGD/R (Figure 5C–F). We also observed reduced ROS level upon DMM treatment in vitro (Figure S3, Supporting Information). We further found inhibition of succinate oxidation by DMM and inhibition of PADs by BB‐Cl could both decrease the percentage of microglia with nuclear decondensation (Figure 5J,K). In addition, DMM and BB‐Cl markedly diminished OGD/R‐induced citH3 and MPO expression, as well as ds‐DNA and MPO releasing in culture medium (Figure 5L–P). Furthermore, the protein levels of citH3, MPO, and MMP9 also decreased by DMM and BB‐Cl when compared with OGD/R group (Figure 5Q,R). In addition, using PAD2‐ and PAD4‐specific inhibitors, we identified PAD2 as a regulator of MiETosis following OGD/R (Figure S5, Supporting Information). Also, SDHA knockdown could attenuate MiETosis induced by OGD/R (Figure S6, Supporting Information).

**Figure 6:**
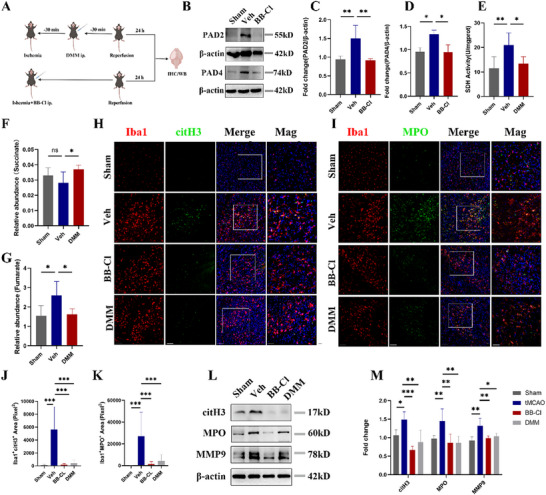
MiETosis was suppressed after inhibition of succinate oxidation and PADs in vivo. A) Schematic illustration of the experimental design framework for this section. B,C) The representative immunoblots and statistical analysis of PAD2, PAD4 and β‐actin in peri‐infarction tissues in Sham, Veh, and BB‐Cl groups (n = 4/group, t test). D) SDH activity in peri‐infarction tissues in Sham, Veh, and DMM groups (n = 6/group, one‐way ANOVA followed by multiple comparisons). The content of E) succinate, F) fumarate in mice treated with DMM (n = 5/group, one‐way ANOVA followed by multiple comparisons). G–K) Representative images of citH3/Iba1 double staining, MPO/Iba1 double‐staining in each group of microglia and quantification analysis of citH3/Iba1 double positive, MPO/Iba1 double positive area (n  =  10–13 images from four animals/group, one‐way ANOVA followed by multiple comparisons) and MPO/Iba1 double positive area (n  =  11–12 images from four animals/group, one‐way ANOVA followed by multiple comparisons). L) Representative bands of citH3, MPO, and MMP9. M) Quantitative analysis of citH3, MPO, and MMP9 protein expression (n  =  4–5/group, one‐way ANOVA followed by multiple comparisons). All data are presented as mean ± SD, *p < 0.05, **p < 0.01, ***p < 0.001.

We further tested whether DMM or BB‐Cl could alleviate MiETosis after cerebral I/R (**Figure** **6**A). First, the effect and efficiency of inhibition of BB‐Cl was verified by western blotting (Figure 6B,C). Besides, 160 mg/kg of DMM could significantly inhibit succinate oxidation shown by the decreased SDH activity, elevated level of succinate, and decreased content of fumarate (Figure 6D–F). Double staining of citH3 and Iba1 displayed high numbers of citH3/ Iba1 double positive cells and area in peri‐infarction area of Veh group mice. Similarly, MPO‐ and Iba1‐ positive area was also high in peri‐infarction area of Veh group mice. DMM and BB‐Cl significantly decreased citH3 and MPO expression (Figure 6G–K). Western blotting further confirmed that DMM and BB‐Cl significantly inhibited the increase in citH3, MPO and MMP9 induced by cerebral I/R (Figure 6L,M). Taken together, these results suggested that SDHA could mediate MiETosis by regulating succinate oxidation and mtROS production.

### 2.6 Inhibition of MiETosis Alleviate Neuronal Injury and Promote Neurological Functional Recovery after Cerebral I/R

**Figure 7:**
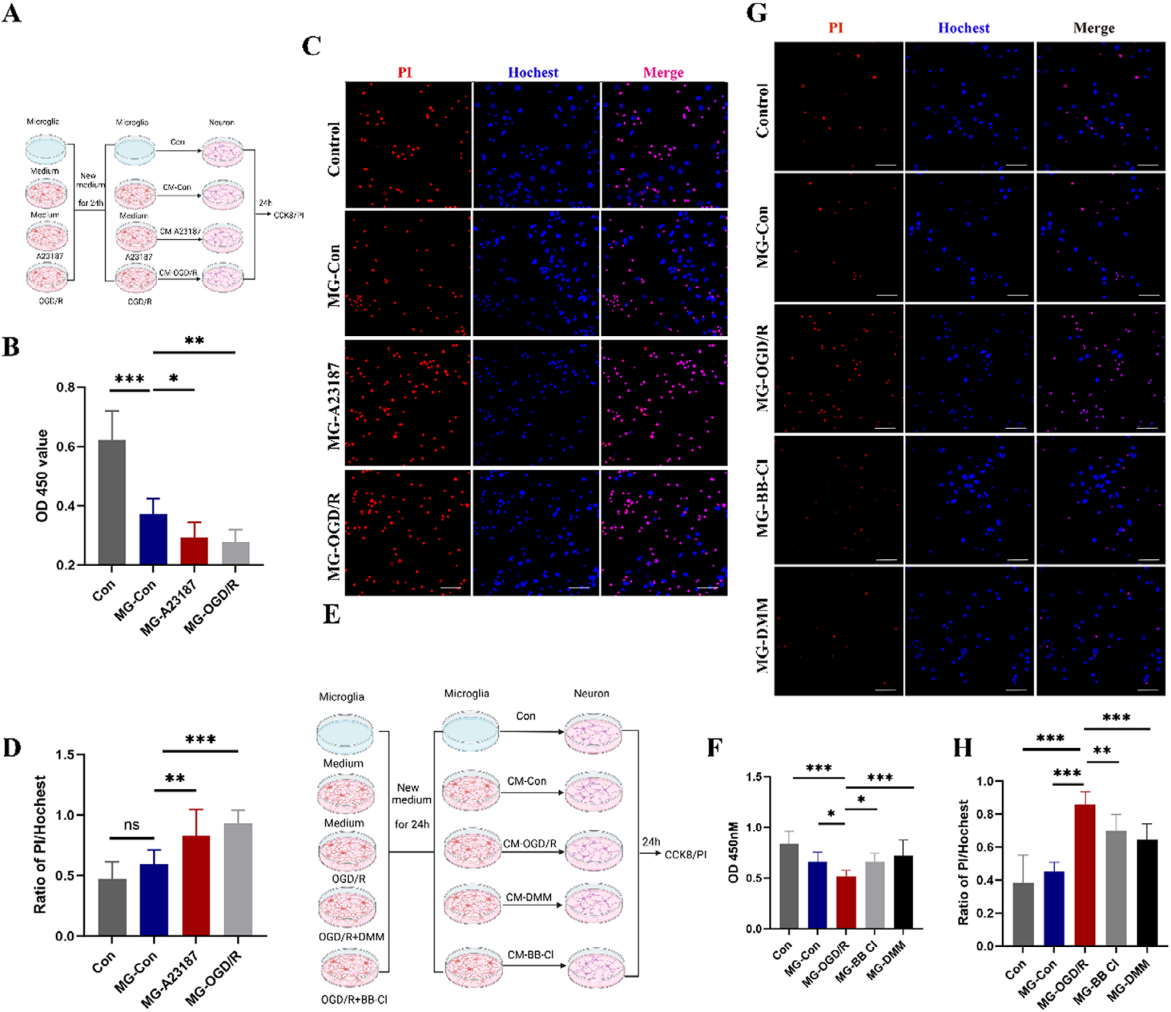
Inhibition of MiETs formation protected neuron from injury in vitro. A,E) Schematic illustration of the experimental design framework for this section. B) Cell viability of neurons was detected by CCK‐8 assay after incubation with supernatant from microglia in Con, A23187 and OGD/R groups. C) PI‐Hoechst double‐stained neurons treated with four types of microglia culture medium. D) Quantification of PI/Hoechst double‐positive cells. Scale bar = 50 µm. F) Cell viability of neurons was detected by CCK‐8 assay after incubation with supernatant from microglia in Con, OGD/R, BB‐Cl, and DMM groups. G) PI‐Hoechst double‐stained neurons treated with five types of microglia culture medium. H) Quantification of PI/Hoechst double‐positive cells in these five groups. Scale bar = 50 µm. All experiments were performed at least three independent times. All data are presented as mean ± SD, *p < 0.05, **p < 0.01, ***p < 0.001.

We further examined the effect of MiETs on neuronal survival by CCK8 and PI/Hoechst staining. As shown in **Figure** **7**A,E, we cultured primary cortical neurons with culture medium of microglia under various treatment. The results suggested that the culture medium of A23187 and OGD/R‐treated microglia significant decreased neuronal activity, and increased ratio of PI‐positive neurons (Figure 7B–D). However, upon treatment with DMM and BB‐Cl, the neurotoxic potential of microglial supernatants was markedly attenuated in comparison to that observed in the OGD/R group (Figure 7F–H), indicating protective effect against neuronal damage by inhibition of MiETosis.

**Figure 8:**
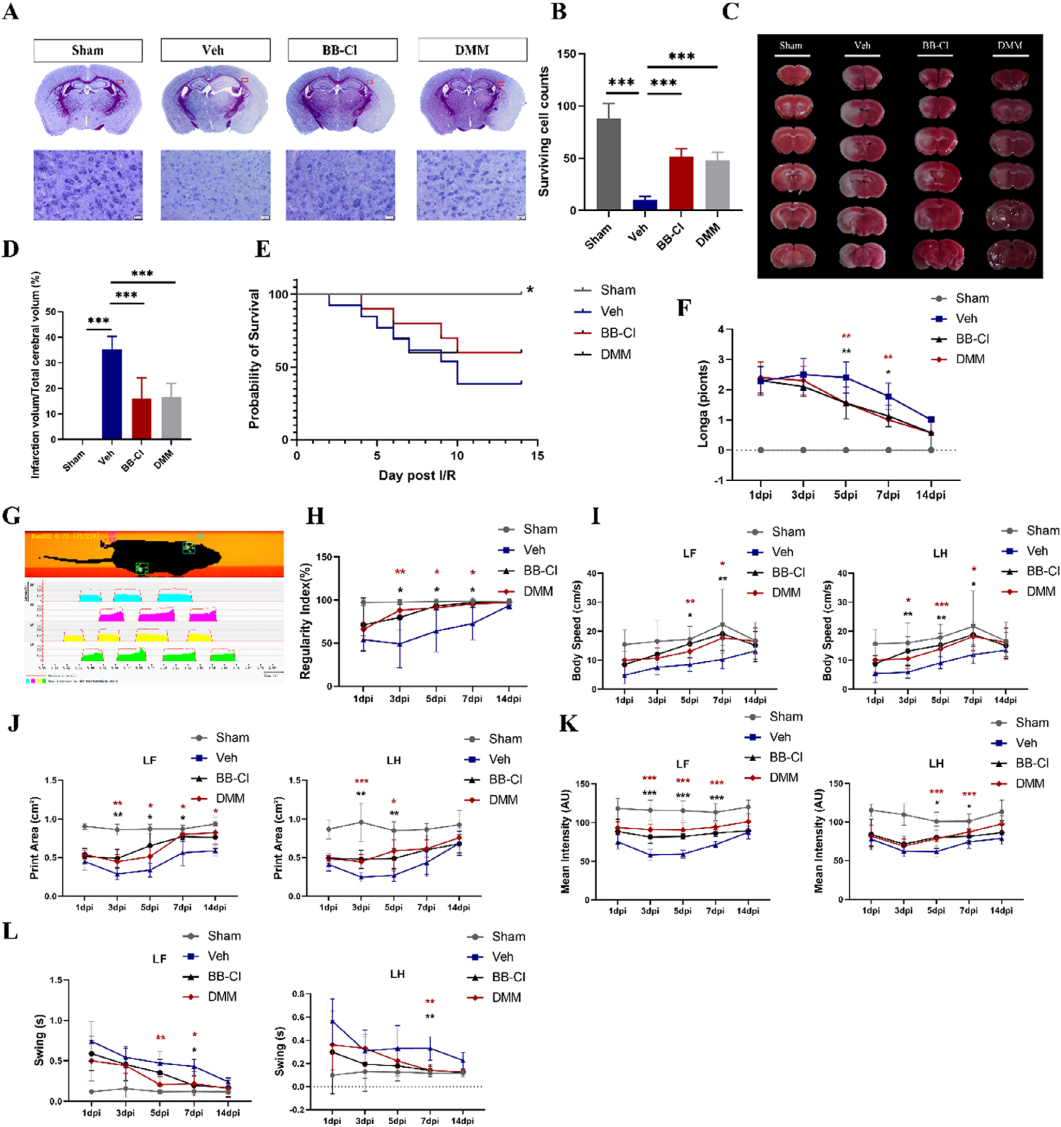
Inhibition of MiETosis by DMM or BB‐Cl alleviate neuronal injury and promote neurological functional recovery after cerebral I/R. A,B) Representative images of Nissl staining and quantification of surviving cells among sham, Veh, DMM, and BB‐Cl groups. Scale bar = 1 mm in the upper panel and scale bar = 20 µm in the lower panel (n  =  21 images from six animals/group, one‐way ANOVA followed by multiple comparisons). C,D) Representative images of TTC staining and quantitative analysis of the percentage of infarct volume relative to total cerebral volume among sham, Veh, DMM, and BB‐Cl groups 24 h after cerebral I/R (n  =  8/group, one‐way ANOVA followed by multiple comparisons). E) Survival curves of sham, Veh, DMM, and BB‐Cl groups during the 14‐day observation period. Mortality data for the sham group was significantly differences from the other groups, *P < 0.05 as determined by Log‐rank (Mantel‐Cox) test. F) Neurological changes were observed by Longa neurological scores among sham, Veh, DMM, and BB‐Cl groups from 1–14 days. G) A diagrammatic sketch marked with footprints and footprint intensity of the sham group. DMM, and BB‐Cl significantly increased H) body regularity index, I) body speed, J) print area, K) mean intensity, and L) swing time of the left forepaw and left hindpaw compared with the Veh group. n  =  10–13/group at the beginning, two‐way ANOVA (Bonferroni's multiple comparison test). LF: left forepaw, LH: left hindpaw. All data are presented as mean ± SD, *p < 0.05, **p < 0.01, ***p < 0.001.

We further studied the influence of inhibiting MiETosis on tissue damage and the recovery of neurological function after I/R injury in mice. The data of Nissl staining revealed a markedly decrease in the number of Nissl bodies scattered throughout the ischemic region in the Veh group compared to the sham group. While DMM and BB‐Cl markedly ameliorated the reduction in Nissl staining and quantitative analysis showed that numbers of surviving cells in DMM and BB‐Cl groups were significantly increased compared with the Veh group (**Figure** **8**A,B). Similarly, enzymatic degradation of MiETs by DNase I could also protect neurons against I/R‐induced injury (Figure S7A, Supporting Information). TTC staining showed that DMM and BB‐Cl could significantly decrease the infarct volume (%) induced by cerebral I/R injury (Figure 8C,D). These results indicated that DMM and BB‐Cl protected against cerebral I/R‐induced neuronal and tissue damage.

Furthermore, we investigated the effect of DMM and BB‐Cl on functional recovery after cerebral I/R. We first observed the change of mortality within 14 days after cerebral I/R (Figure 8E). The results show that 100% of sham group mice survived within 14 days, while only 76.9%% of mice survived for 5 days after cerebral I/R, compared with 90.0% of mice in the DMM group and 90.0% of mice in the BB‐Cl group. At 14 days, survival rates dropped to 38.5%, 60.0%, and 60.0% in I/R, DMM, and BB‐Cl groups, respectively. Accordingly, DMM and BB‐Cl could reduce mortality after cerebral I/R. Longa scores showed significantly better neurological recovery in DMM and BB‐Cl groups compared with the Veh group at 5–7 days after cerebral I/R (Figure 8F). We also performed gait analysis with the CatWalk system after cerebral I/R to objectively evaluate the effect of DMM and BB‐Cl (Figure 8G). Animals in DMM and BB‐Cl groups displayed significant increases in regularity index (Figure 8H), body speed (Figure 8I), and paw pressure (including print area, mean intensity, max contact area, max contact mean intensity, and max intensity) (Figure 8J,K and Figure S7B–D, Supporting Information), as well as significant decreases in swing time (Figure 8L) of the left forepaw and left hindpaw compared with the Veh group. Taken together, these results confirm that inhibition of SDHA significantly ameliorated neurological functional defects and gait disturbance after cerebral I/R.

## 3 Discussion

Previous researches had demonstrated several different functional phenotypes of microglia participate in the process of neuronal damage after IS.^[^ ^35^ ^]^ In this study, we aimed to explore the role and mechanism of succinate dehydrogenase (SDH)‐mediated succinate oxidation in microglia extracellular traps (MiETs) formation and neuronal injury after cerebral I/R injury. We demonstrated that cerebral I/R and OGD/R could induce MiETosis, and as well as enhanced glycolysis, SDH activity, high expression of SDHA and succinate oxidation in microglia during reperfusion. Our data further showed that inhibiting SDHA could reduce MiETosis by suppressing succinate oxidation and mtROS production during reperfusion. Furthermore, SDHA inhibition was found to alleviate neuronal injury and neurological behavior disorders after I/R injury. The result of this study indicated that microglia are the main source of ETs in the peri‐infarction area at the first 24 h after cerebral I/R injury, which is similar to previous result from Arnaud Mansart et al.^[^ ^15^ ^]^ They found that CD11b^+^CD45^+^citH3^+^/MPO^+^ MiETs could be observed within spinal cord tissue 1 day after spinal cord injury. And MiETs were primarily distributed within a range of 300 µm from the injury center, which is generally consistent with the range of the peri‐infarction region defined in cerebral I/R, which is ≈500 µm around the ischemic core.^[^ ^36^ ^]^ Although there is no study observed the percentage of NETs and MiETs at 24 h, we can get some supportive evidences from the limited number of infiltrated neutrophils into cerebral parenchyma following reperfusion. A single‐cell transcriptomic analysis of the immune cell landscape in the mouse brain after tMCAO reveals that the number of microglia is far more than neutrophils at 24 h after tMCAO.^[^ ^37^ ^]^ Our neutrophil depletion experiments further confirmed this, demonstrating that neutrophils contribute minimally to parenchymal ETs. Kang et al. reported that neutrophils is the main source of ETosis shown by citH3 positive signal at 72 h after tMCAO,^[^ ^38^ ^]^ we proposed that the differences might be due to the distinct parameters for tMCAO model establishment. It has been demonstrated that neutrophils are scattered mainly inside vascular rather than parenchyma at the first day post cerebral I/R injury.^[^ ^39^, ^40^ ^]^ The predominant localization of MiETs within the peri‐ischemic region suggests a potential for direct contact and immediate effect of microglia on neuronal survival. Given all that mentioned above, MiETs represent an additional, significant neural injury mechanism that merits attention and cannot be overlooked.

Cells undergoing suicidal ETosis immediately die while forming ETs, whereas cells following vital ETosis survive and continue to function normally.^[^ ^41^ ^]^ We performed CCK8 to investigate the effect of OGD/R on microglia activity which implies that OGD/R induce MiETs formation leading to cell death. Whether there are vital MiETosis and how long microglia can survive after expelling their DNA are still unclear and need further investigation.

Listeria‐mediated MiETs was reported to be cytosolic ROS‐ and NADPH oxidase (NOX)‐dependent,^[^ ^16^ ^]^ while dopamine‐induced MiET formation is independent of ROS.^[^ ^17^ ^]^ This discrepancy could be attributed to pathways involved in ETs formation is highly stimulus dependent. Instead of ROS, many current investigations into the underlying mechanisms of ETosis consistently underscore the pivotal function of mtROS in this process.^[^ ^42^, ^43^, ^44^ ^]^ Mitochondrial ROS scavengers was also reported to diminish mtROS levels in neutrophils and subsequently suppress NETosis.^[^ ^45^ ^]^ In addition, preincubation with the mitochondrial uncoupler dinitrophenol (DNP) abolished ionomycin‐induced mitochondrial ROS production and MiET release in a dose‐dependent manner.^[^ ^16^ ^]^ In this study, we found that mtROS is involved in METosis. However, we also observed increased cytosolic ROS after OGD/R treatment and the level of ROS was decreased after SDH inhibition (Figure S3, Supporting Information). A possible explanation for this might be that mtROS constitutes a subset of cytosolic ROS, implying that fluctuations in their levels are synchronized. Besides, previous study indicated that mtROS can stimulate the NOX in vascular endothelium in vivo which result in increased cytosolic ROS.^[^ ^46^, ^47^ ^]^ Thus, it could not be excluded that the contribution of glycolysis such as pentose phosphate (PPP) pathway to ROS. Further investigations are still needed to figure out its role in MiETosis though the inhibition of mtROS was sufficient to limit MiETs formation in our experiment.

Subsequently, we utilized metabolite detection and other methods to determine the origin of ROS/mtROS. Our results indicated that during cerebral ischemia, the levels of most glucose metabolites decreased due to insufficient substrate availability, whereas succinate accumulated in brain tissue and declined after reperfusion. This observation is consistent with the work conducted by Thomas Krieg et al., who observed the accumulation of succinate during ischemia and its oxidation during reperfusion in both mouse and human brain tissues through mass spectrometry imaging.^[^ ^48^ ^]^ The accumulation of succinate is a hallmark of ischemia^[^ ^24^, ^49^, ^50^ ^]^ and is proposed to occur via the reversal of activity of SDH driven by a highly reduced Co‐Q pool.,^[^ ^24^, ^51^ ^]^ and/or canonical Krebs’ cycle activity.^[^ ^52^ ^]^ Upon reperfusion, SDH rapidly oxidizes succinate back to fumarate, driving RET and generating mtROS.^[^ ^53^ ^]^ This underscores the crucial role of SDH and succinate oxidation in the intricate pathophysiology of I/R injury. Furthermore, our investigation unveiled that SDHA, a pivotal subunit of SDH regulating succinate metabolism following cerebral I/R, is predominantly overexpressed in microglia. This evidence implies that microglia may serve as the primary source of mtROS generation post‐I/R, potentially triggering MiETosis within these cells. Our in vitro experiments further confirmed this hypothesis.

The role of succinate oxidation in MiETosis was further confirmed by the results that DMM administration during reperfusion significantly decreased SDH activity in I/R brain tissue and microglia, reduced mtROS levels and diminished the occurrence of MiETosis. The protective effect of succinate oxidation inhibition was proved by several researches which demonstrated that administering SDH inhibitors during reperfusion can inhibit the production of mtROS by suppressing succinate oxidation in cardiomyocytes and kidney, thereby protecting these organs during I/R injury.^[^ ^24^, ^34^, ^54^, ^55^ ^]^However, there is only one study examined the effect of SDH inhibition on NETs formation which displayed that 10 mM DMM could significantly reduce mtROS production and diminish NET formation when neutrophils were cultured with *T. gondii*.^[^ ^56^ ^]^ Despite of different stimuli and cell type, this study still provides support for our findings.

We further found that the supernatant of microglia treated with OGD/R and A23187 could damage primary neurons. After inhibiting MiETosis with DMM and BB‐Cl, the effect of their supernatant on inducing neuronal death was significantly reduced, suggesting that the occurrence of MiETosis may be one of the mechanisms of neurological injury after cerebral I/R. In vivo experimental results showed that DMM treatment significantly reduced neuronal death in the ischemic area, reduced infarct volume, and promoted neurological functional recovery in mice after cerebral I/R mice. These indicates that targeting MiETosis is beneficial for reducing cerebral I/R injury. Currently, most studies suggest that ETs possess tissue and cell toxicity, as evidenced by their involvement in thrombosis,^[^ ^57^ ^]^ acute kidney injury,^[^ ^58^ ^]^ acute lung injury,^[^ ^59^ ^]^ asthma,^[^ ^60^ ^]^ promoting the onset and progression of atherosclerosis,^[^ ^61^ ^]^ and delayed wound healing.^[^ ^62^ ^]^ This is primarily related to their mechanisms of occurrence and components. Besides ETosis, ROS/mtROS themselves can cause cellular and tissue damage.^[^ ^63^ ^]^ Furthermore, several components of ETs, including DNA,^[^ ^64^ ^]^ myeloperoxidase (MPO)^[^ ^65^ ^]^ and histones,^[^ ^66^ ^]^ are cytotoxic. Therefore, inhibiting the occurrence of ETosis or eliminating ETs can both alleviate cellular and tissue damage to a certain extent. For instance, Cl‐amidine (a PADs inhibitor) can reduce liver I/R injury by decreasing macrophage extracellular traps.^[^ ^67^ ^]^ Additionally, administration of DNase I to degrade eosinophils extracellular traps (EETs) in the airways of patients with allergic asthma can reduce the viscosity of their sputum and prevent disease progression.^[^ ^60^ ^]^

The current investigation was constrained by certain limitations. One limitation of the study was that we use immunofluorescence to validate the contribution of microglia to ETosis. Other technology like flow cytometry, intravital imaging may further strengthen the importance of MiETs. Furthermore, our investigation demonstrated that succinate oxidation, dependent on SDH, significantly promoted MiETosis in the microglia during I/R, thereby leading to neurological injury. However, the possibility of other mechanisms involved in MiETosis, such as calcium overload, endoplasmic reticulum stress et al. induced by ischemia‐reperfusion injury, cannot be excluded. It has been reported that calcium influx could activate PADs and promote extracellular trap (ET) formation.^[^ ^68^ ^]^ Nevertheless, the underlying mechanism for calcium influx still remains elusive. Our result of immune electron microscopy showed that gold particles of Iba1 mainly translocated into euchromatic region of nucleus of microglia after cerebral ischemic injury. As a calcium‐binding protein, involvement of Iba1 in regulating nuclear calcium signal during MiETosis deserves further exploration.

In conclusion, our findings show that succinate oxidation mediated MiETosis act as an important mechanism of cerebral IR injury. Moreover, the rapid oxidation of succinate by SDH may be inhibited by the administration of DMM on reperfusion, leading to a reduction in mitochondrial ROS production and the occurrence of MiETosis which may be a novel strategy for treatment of ischemic stroke.

## 4 Experimental Section

### Animals and Experimental Protocol

Adult male C57BL/6 mice (20–25 g, 6–8 weeks) and Sprague‐Dawley rats (250–300 g, 6–8 weeks) were housed in a pathogen‐free facility with a 12 h light/dark cycle and allowed free access to food and water. Rats were randomly assigned to the middle cerebral artery occlusion (MCAO) group or sham group and underwent metabolomics analysis after surgery.

Dimethyl malonate (DMM) or BB‐Cl‐Amidine hydrochloride (BB‐Cl) to assess the influence of succinate oxidization and MiETosis on cerebral I/R damage in mice. DMM (D806604, Macklin, Shanghai, China) and BB‐Cl (HY‐111347A, MedChemExpress, Monmouth Junction, NJ, USA) were dissolved in a vehicle buffer (Veh) containing saline, 1% dimethyl sulfoxide (DMSO, D2650, Sigma‐Aldrich) and the final volume is 200ul. DMM was injected intraperitoneally (i.p.) at a dosage of 160 mg kg^−1^, 30 min before reperfusion, as previously described.^[^ ^34^, ^54^ ^]^ BB‐Cl was administered i.p. at a dosage of 1 mg kg^−1^, 30 min before surgery.^[^ ^69^, ^70^ ^]^ Mice were randomly divided into five groups: (1) sham group mice underwent surgery without transient MCAO (tMCAO), (2) tMCAO group mice received tMCAO, (3) Veh group mice underwent tMCAO and received Veh, (4) DMM group mice underwent tMCAO and received DMM, and (5) BB‐Cl group mice underwent tMCAO and received BB‐Cl. Ly6G^+^ cell depletion was induced with anti‐Ly6G mAb clone 1A8 (BioXcell, USA) injected at 0.5 mg i.p. on days –3, –2, and –1 relative to model establishment as previously described.^[^ ^71^ ^]^DNase‐I (50 µg in 250 µL of normal saline i.p. and 10 µg in 250 µL of normal saline as a second dose intravenously) or the vehicle was injected into mice bodies 3 h after tMCAO induction, as the previous research described.^[^ ^72^ ^]^ All animal experiments were approved by the Committee on the Ethics of Animal Experiments of the College of Medicine, Xi'an Jiaotong University and followed National Research Council Guidelines (No. 2019–218).

### MCAO and tMCAO Model

MCAO and cerebral I/R models were established as previously described.^[^ ^73^, ^74^ ^]^ Rats or mice were anesthetized with isoflurane (5% for induction and 1.5% for maintenance) and their core temperature maintained at 36.0 ± 0.5 °C throughout the procedure using a homeothermic heat mat (GE0‐20W, GLOBALEBIO, Beijing, China). A silicon rubber‐coated nylon monofilament (3600, Guangzhou Jialing Biotechnology, Guangzhou, China) is inserted into the middle cerebral artery (MCA) via right internal carotid artery (ICA) in rats to establish MCAO model. For tMCAO model, a 3‐0 nylon monofilament wire (MSMC21B120PK50, RWD Life Science, Shenzhen, China) was inserted from the right external carotid artery into the right ICA and advanced MCA in mice. At 60 min after MCAO, the brain was reperfused by the withdrawal of the intraluminal suture. And the sham group underwent the same procedures except for insertion of the monofilament. When animals fully awake after surgery, their neurological deficiency were initially examined according to Longa's five‐tiered grading system. Animals with 1–3 points of Longa score were considered successful models.^[^ ^75^ ^]^

### Tissue Sample Preparation

1% pentobarbital sodium (100 mg kg^−1^. i.p.) was used to anesthetize animals at the indicated time points. For tissue sampling, tissue in ischemic border was rapidly separated and placed in Eppendorf tubes which was stored at −80 °C. The peri‐infarction area was defined as the ≈500‐µm‐wide region that surrounds the core.^[^ ^76^ ^]^ To prepare frozen section, mice were first subjected to phosphate‐buffered saline (PBS, 0.01 M) transcardiac perfusion followed by 40 mL of 4% paraformaldehyde (PFA). Next, brains were removed and postfixed in 4% PFA, followed by dehydration in 30% sucrose. After embedding in OCT compound (4583, Sakura, CA, USA). After a quick freeze and fixation, brains were sectioned (Leica CM1950 Cryostat, Wetzlar, Germany) at the thickness of 20 um.

### LC‐MS and GC‐MS Analysis of Metabolomics

Rat brain samples were harvested 4.5 h after operation in each of the MCAO and sham groups (six rats per group). Liquid chromatography‐mass spectrometry (LC‐MS) and gas chromatography‐mass spectrometry (GC‐MS) were performed as we previously described.^[^ ^77^, ^78^ ^]^

### Extraction of Metabolites

Mouse brain samples were harvest 24 h after surgery in each group, weighed on dry ice and placed in pre‐chilled Eppendorf tube (six mice per group). Next, 1.5 mL extraction buffer (50% [v/v] methanol, 50% [v/v] ultrapure water, was added to the Eppendorf tubes and the tissue was homogenized using a tissue homogenizer (25Hz, 5 min; Thermo Fisher Scientific, Waltham, MA, USA). Samples were then centrifuged at 16,000 g at 4 °C for 10 min and the supernatant was collected and the supernatant was transferred to new pre‐chilled Eppendorf tube and were stored at −80 °C until quantification of metabolites by liquid chromatography‐tandem mass spectrometry (LC‐MS/MS).

### LC‐MS/MS Quantification of Succinate and other TCA Metabolites

To quantify succinate and other TCA metabolites a LCMS‐8060 mass spectrometer (Shimadzu, UK) with a Nexera X2 UHPLC system (Shimadzu, UK) was used as described before.^[^ ^79^ ^]^ Briefly, metabolites were separated using a SeQuant ZIC‐HILIC column (3.5 um, 100 Å, 150 × 2.1 mm, 30 °C column temperature; MerckMillipore, UK) with a ZIC‐HILIC guard column (200 Å, 1 × 5 mm) at a flow rate of 200 µL min^−1^ with mobile phases of A) 10 mM ammonium bicarbonate and B) 100% acetonitrile. The MS was operated in negative ion mode with multiple reaction monitoring (MRM) and spectra acquired using Labsolutions software (Shimadzu, UK). Succinate and malonate were quantified using standard curves relative to succinate, fumarate, malonate, α‐ketoglutarate and oxaloacetate internal standard.

### Real‐Time Quantitative Polymerase Chain Reaction

Total RNA was extracted and purified from cultured cells or tissues using TRizol (15596018, Life Technologies, Waltham, MA, USA). Then, the RNA of each group was reverse transcribed into cDNA using PrimeScriptTM RT reagent Kit (RR036A‐1, Takara, Kusatsu, Japan). Quantitative RT‐qPCR was performed by a Bio‐Rad CFX system (Hercules, CA, USA) using SYBR Green Master Mix (RR820A, Takara). All gene expression values were normalized to β‐actin mRNA levels in each sample using the 2^−∆∆Ct^ method. Primer sequences are listed in Table S1, Supporting Information.

### Western Blot

Brain tissues and cell were lysed in RIPA buffer (Beyotime, Shanghai, China) containing protease inhibitor (MilliporeSigma, Billerica, MA, America) and phosphatase inhibitor (MilliporeSigma, Billerica, MA, America). The total protein concentration in supernatant was determined by a BCA protein assay kit (Beyotime, Shanghai, China). Aliquots of homogenate with equal protein concentration were separated by sodium dodecyl sulfate‐polyacrylamide gel and then transferred to polyvinyl difluoramine membrane. After blocking in 5% non‐fat milk for 2 h at room temperature, the membranes were then incubated with primary antibodies overnight at 4 °C, including: citrullinated histone h3 (citH3, ab5103, 1:1000, Abcam, Cambridge, UK), MPO (AF3667, 5 µg mL^−1^, R&D, Minnesota, USA), MMP9 (GTX100458, 1:1000, GeneTex, Irving, California, USA), PAD2 (12110‐1‐AP, 1:1000, Protientec, Rosemont, IL, USA), PAD4 (DF6685, 1:1000, Affinity, Jiangsu, China), glucose transporter type 1 (GLUT1, ab115730, 1:10 000, Abcam), pyruvate kinase isozyme type M2 (PKM2, 25659‐1‐AP, 1:5000, Proteintec), 6‐Phosphofructo‐2‐Kinase (PFKFB3, 13763‐1‐AP, 1:1000, Proteintec), SDHA (ab14715, 1:10 000, Abcam) and β‐actin (A5441, 1:10 000, Sigma‐Aldrich) After incubation with appropriate horseradish peroxidase‐conjugated secondary antibodies (31458, SA1‐100, PA1‐28664, 1:2000, Thermo Fihser Scientific) for 2 h, bands were developed and visualized using VisionWorks (Analytik Jena, Jena, Germany). The results were analyzed with ImageJ software (National Institutes of Health, Bethesda, MD, USA).

### Immunohistochemistry

Brain sections were washed with PBS followed by blocking with blocking solution (10% donkey serum, 0.5% bovine serum albumin (BSA), and 0.25% Triton X‐100 in PBS) for 1 h, then incubated with the primary antibody at 4 °C overnight. The following primary antibodies and dilutions were used: ionized calcium binding adaptor molecule 1 (Iba1, 011–27991/019‐19741, 1:500, Wako, Tokyo, Japan), neuronal nuclei (OB‐PRT013, 1:500, Oasisbiofarm, Las Vegas, NV, USA), glial fibrillary acidic protein (GFAP, ab4674, 1:300, Abcam), lymphocyte antigen 6 family member G (Ly6G, ab25377, 1:400, Abcam), Ly6G/C (14‐5931‐82, 1:300, Invitrogen), citH3 (1:400), MPO (ab25989, 1:300, Abcam), SDHA (1:1000). The brain sections were incubated with corresponding Alexa Fluor 594‐, Alexa Fluor 488‐, or Alexa Fluor 647‐labeled secondary antibodies for 2 h at room temperature in the dark the next day. The nuclei were counterstained with 4’,6‐diamidino‐2‐phenylindole (DAPI, D9564, Sigma‐Aldrich) for 10 min. Then slides were washed three times with PBS and covered using 50% glycerol in PBS. Confocal fluorescence microscopy (Olympus FV3000, Tokyo, Japan and VS200, Olympus, Tokyo, Japan) was used to capture images. ImageJ software was used to quantitatively analyzed images. The image processing software Imaris was used to reconstruct 3D images and quantitatively analyzed. Sections from four animals in each group were immunohistochemically stained and used for double‐ or triple‐labeling experiments.

### Measurement of SDH, LDH and ATP

To quantify activity of SDH, lactate dehydrogenase (LDH), and level of adenosine 5'‐triphosphate (ATP), tissues, and cells were collected and assayed with kits according to the manufacturer's instructions. Samples were homogenized after reperfusion or culture. After centrifuging at 4 °C and 1.2 × 10^4^ g for 10 min, the supernatant of samples was used to detect activity of SDH (BC0950, Solarbio, Beijing, China), LDH (A020‐2‐2, Nanjing Jiancheng Bioengineering Institute, Nanjing, China), and ATP levels (S0027; Beyotime Biotechnology, Haimen, China). Besides, the total protein concentration was determined by a BCA protein assay kit and used to standardize levels of SDH, LDH and ATP.

### Primary Microglia Culture

Primary microglia were isolated and purified from 1‐2‐day‐old C57BL/ 6J mice as described previously.^[^ ^80^ ^]^ In brief, brain membrane was removed and cerebral cortex was digested with 0.25% tripsin EDTA for 10 min. Then, we added the same amount of DMEM medium containing 10% FBS to terminate the digestion. The cell pellet was resuspended in high glucose Dulbecco's Modified Eagle Medium (DMEM) supplemented with 100 IU mL^−1^ penicillin, 100 ug mL^−1^ streptomycin, and 10% fetal bovine serum (FBS). Cells were seeded in a 75‐cm^2^ culture flask and maintained at 37 °C in a humidified incubator with 5% CO_2_. At the third day, the culture medium was changed. Microglia was harvest from cultured medium at about 10–12 day when microglia became matured and floated in the medium. The primary microglia were cultured in 90% DMEM, 10% FBS, 100 IU mL^−1^ penicillin, 100 ug mL^−1^ streptomycin at 37 °C in a humidified atmosphere of 5% CO_2_.

### OGD/R and Drugs Treatment

OGD experiments were performed using an incubator (Theremofisher Scientific) with a premixed gas (95% N_2_, 5% CO_2_) kept at 37 °C. Briefly, microglia were cultured with glucose‐free medium and was placed into the hypoxia chamber for 4 h. After OGD, the cells were perfused by DMEM with glucose and transferred to a 5% CO_2_‐95% O_2_ air incubator for a relative amount of time. A23187 (10 mM, 52665‐69‐7; MedChemExpress) stock solutions were dissolved in DMSO at a storage concentration. This reagent was added into the medium for 4  h at 5  uM to induce MiETosis in vitro. Cells were treated with BB‐Cl (2  uM, dissolved in DMSO), GSK484 (HY‐100514, 5 uM, MedChemExpress), PAD‐IN‐2/AFM32a (HY‐136557, 5 uM, MedChemExpress) at the same time with OGD/R. DMM (20 mM, dissolved in DMSO) and 2‐DG (HY‐13966, 0.5 mM, MedChemExpress) was added when microglia were perfused with DMEM and oxygen. The control group was cultured in medium containing 0.004% DMSO for the same time with other groups.

### Cell Viability

Microglia were seeded onto 96‐well plates (6 × 10^3^ cells per well), and their viability was assessed after treatment using a Cell Counting Kit‐8 (CCK‐8) assay (IC1519, InCellGene, USA). Briefly, 10 uL of reagent was added into 100 uL medium and incubated with cells at 37 °C for 1 h and then the absorbance was measured at 450 nm by a multimode microplate reader (Tecan Infinite M200). As for the viability of neurons detection, after treatment the medium was discard and new medium was added into the plates. After another 24 h, the culture supernatant was collected and added into the medium of neurons and cultured for 24 h. Then CCK‐8 test was performed.

### Immunocytochemistry

Cells were grown on cell glass slides, rinsed with PBS, fixed with 4% PFA, and washed with PBS for three times. Next, the slides were blocked with 3% BSA with 0.3% Triton X‐100 for 1 h at room temperature, and incubated with the following primary antibodies overnight at 4 °C: Iba1, citH3, MPO (ab90810,1:1000, Abcam), and SDHA. After washing slides with PBS for three times, they were incubated with corresponding secondary antibodies (1 h) and DAPI (5 min) in the dark at room temperature. Finally, slides were mounted with glycerol and imaged using a confocal microscope (Olympus FV 3000).

### Scanning Electron Microscopy

Microglia was collected after treatment and then was treated with fixation, dehydration, mounting & coating and finally image acquisition.

### Imaging Flow Cytometry of Hoechst and MitoSOX

Cells were seeded onto 6‐well plates (1 × 10^5^ per well). Following all interventions, microglia was first washed with PBS for two times, and incubated with Hoechst 33342 (5 ug mL^−1^, C1011, Beyotime) for 10 min or mitoSOX (0.5 uM, M36009, Thermo Fisher Scientific) for 30 min in dark in 37 °C. Microglia was washed with PBS for three times and digested with 0.25% tripsin EDTA for 5 min. We added the same amount of DMEM medium containing 10% FBS to terminate the digestion. The cell pellet was resuspended in pre‐chilled PBS and analyzed on an Amnis ImageStreamX (Luminex, Austin, TX, USA) flow cytometer.^[^ ^81^, ^82^ ^]^ Imaging flow cytometry with a 405 nm laser (200 mW) was used for detection, while channel with emission wavelength of 450 nm (Ch09) and 610 nm (Ch05) were used to collection and analyzation. The sample flow rate was set to medium with 40 × magnification. Single cells were gated based on Area_M01 versus Aspect Ratio _M01 (i.e., size vs. circularity). A region (R1) was created to gate for a single population. At least 1 × 10^4^ events were recorded in R1 for every sample. Collected data were analyzed using ImageStream Data Exploration and Analysis Software (Luminex). The results of flow cytometric analysis of Hoechst (Ch09) and mitoSOX are expressed in mean area and mean fluorescence intensity (MFI).

### Measurement of ECAR and OCR

Cells were plated in plates provided by manufacture at 2 × 10^5^ cells well^−1^ and then were treated with different reperfusion time. Kits of ECAR (E‐BC‐F069, Elabscience) and OCR (E‐BC‐F068, Elabscience) was used to detect the change of ECAR and OCR of microglia at different time point according to the manufacturer's instruction. Cells were maintained at 37 °C in normal growth medium without serum during tests.

### Measurement of Cytosolic ROS with DCFH‐DA Staining

Following all interventions, microglia grown in confocal dishes were stained with 10 uM DCFH‐DA (S0033S, Beyotime) for 30 min at 37 °C. Cytosolic ROS levels in primary microglia were analyzed using a confocal microscope (Olympus FV 3000) and were assessed as MFI of the 488‐nm excitation channel.

### PI/Hoechst Staining

Cells were grown in confocal dishes and treated with different reagents as described above. After washing cells with PBS, Hoechst 33342 and PI were added to the culture medium at final concentrations of 2 and 5 ug mL^−1^, respectively, and cells were incubated at 37 °C for 15 min. Next, cells were washed and immediately observed and imaged with a confocal microscope (Olympus FV 3000). Finally, the rate of cell death was calculated by determining the PI^+^/Hoechst^+^ ratio.

### 2‐NBDG Uptake

Primary microglia were seeded into 96 well plates overnight. After treatment, replace the medium with a colorless and glucose‐free medium containing 100 ug mL^−1^ 2‐NBDG (see the key resources table). And the relative fluorescence intensities of the 0th and 30th min of each well were recognized by microplate reader. The difference in fluorescence intensities between the two time points was calculated as an index of microglial glucose uptake ability.

### Lentiviral Transduction

The sh‐SDHA‐GFP lentivirus targeting the mouse SDHA was prepared by OBiO Technology Corp.,Ltd (Shanghai, China). Lentivirus expressing scramble shRNA (vector) was used as a negative control. For lentivirus transduction, primary microglia were seeded on plates or dishes as above. The next days post seeding, the medium was removed and added the fresh medium containing sh‐SDHA or vector lentivirus at a certain multiplicity of infection (MOI). 18 h post‐transduction, the medium containing lentivirus was replaced with a fresh culture medium. Transduced microglias were incubated for 3 days and then harvested to detect the transduction efficiency. The efficiency of Lenti‐sh‐SDHA was validated by qRT‐PCR and Western blotting. Finally, based on the pre‐experimental results, an MOI of 60 was selected to continue transduction and used for subsequent observation and analysis. The sequences of shRNA were provided in Table S2, Supporting Information.

### Assessment of Neurological Deficits

Longa score was used to evaluate the neurological functions of mice in each group at 1, 3, 5, 7 and 14 days after reperfusion.^[^ ^83^ ^]^ Assessments were performed by a blinded observer and then confirmed by another blinded observer.

### CatWalk XT Gait Test

To assess functional impairment and recovery, mice underwent a CatWalk XT automated quantitative gait analysis (CatWalk XT, Noldus, Wageningen, Netherlands) at 1, 3, 5, 7, and 14 days after reperfusion, and were trained 3 days before surgery as previously described. Briefly, mice paw prints were captured by a highspeed camera set underneath a long narrow walkway when the mice passed. Mice always traversed from left to right and crossed the walkway to reach their home‐cage which was putted in a dark chamber. Three consecutive trials were performed for each animal. Images from each trial were digitized and analyzed by Catwalk‐XT software (Noldus).

### Infarct Volume Assessment

The infarct volume was detected by using 2,3,5‐Triphenyltetrazolium chloride (TTC, T8877, Sigma‐Aldrich). Briefly, mice were anesthetized and their fresh brains were removed at 24 h after reperfusion. Subsequently, each brain was cut into slices of 1mm and incubated with 1% TTC at 37 °C for 10 min and then fixed in 4% PFA for 30 min. The percentage of infarct volume was calculated according to the formula: cerebral infarction volume/total cerebral volume × 100%.

### Nissl Staining

Nissl staining solution (C0117, Beyotime) was used to stain the Nissl body of the brain slices according to the manufacturer's instruction. A bluish‐purple color was observed to display the basic nervous structure of the brain. Histological changes were observed with a VS200 microscope (Olympus) to assess neuronal damage and ImageJ software was used to analyze stained cells.

### Transmission Electron Microscopy and Immunodetection

At 24 h after reperfusion, mice (n = 3 each group) were killed and brain tissue samples were fixed for 1 h at 4 °C in 0.05% (v/v) glutaraldehyde and 2% (v/v) PFA, rinsed in 0.1 mol L^−1^ phosphate buffer (pH 7.4), then post‐fixed for 24 h at 4 °C. Ultrathin sections (100 um) of peri‐infarction region were made with a vibratome (Leica, Wetzlar, Germany) and blocked by 5% BSA and 0.05% Triton‐X at room temperature for 3 h. Ultrathin brain sections were incubated with the anti‐Iba1 antibodies (Wako, Japan) at a 1:300 dilution. After repetitive rinses, they were incubated overnight at room temperature with gold‐labeled (15 nm) secondary anti‐mouse IgG (Amersham, UK) diluted 1:50. Then the slices were putted into silver nitrate solution and incubated in dark for 15 min. After repetitive rinses, the slices were putted into Osmic acid for 2 h and dehydrated in a graded series of ethanol (50%, 70%, 80%, 90%, 95%, and 100%). After staining samples with uranyl acetate and lead citrate, images were taken using an electron microscope (JEM‐1230; JEOL, Tokyo, Japan).

### Statistics

All statistical analyses were performed using GraphPad Prism 9 software (GraphPad Software, San Diego, CA, USA). Data are presented as the mean ± standard deviation (SD). Independent sample two‐tailed Student's *t‐*test and Wilcoxon tests were used to analyze the two groups. For multiple comparisons, one‐way ANOVA was performed, followed by Tukey's post hoc test. Assessment of neurological scores and gait test results were analyzed by two‐way repeated measures ANOVA. Cumulative survival rates were compared among the four groups using the Kaplan–Meier method and log‐rank tests. All experiments were performed at least three independent times. A value of *p* < 0.05 was used to indicate statistical significance.

## Conflict of Interest

The authors declare no conflict of interest.

## Author Contributions

Z.L.L., L.T., and D.M.J. contributed equally to this work and share first authorship. Study Conceptualization: Z.G.L., F.H., K.F., and Z.L.L; Methodology: Z.L.L., L.T., F.H., and K.F.; Formal analysis and investigation: Z.L.L., D.M.J., L.Y., L.J.L., L.Z.W., H.Q., C.Z.Y., and C.Y.J.; Writing‐original draft preparation: Z.L.L.; Writing‐review and editing: Z.G.L., F.H., K.F. and L.T.; Funding acquisition: Z.G.L., F.H., K.F.; Resources: Y.Y., F.Y.X., W.X.Y., J.Y.T., W.H.Y., and W.S.X.; Supervision: L.T., Z.L., J.Y., F.S.H. and G.Y.Y. All authors read and approved the final paper.

## Supporting information



Supporting Information

Supporting Information

## Data Availability

The data that support the findings of this study are available from the corresponding author upon reasonable request.

## References

[1] S. S. Virani , A. Alonso , H. J. Aparicio , E. J. Benjamin , M. S. Bittencourt , C. W. Callaway , A. P. Carson , A. M. Chamberlain , S. Cheng , F. N. Delling , M. S. V. Elkind , K. R. Evenson , J. F. Ferguson , D. K. Gupta , S. S. Khan , B. M. Kissela , K. L. Knutson , C. D. Lee , T. T. Lewis , J. Liu , M. S. Loop , P. L. Lutsey , J. Ma , J. Mackey , S. S. Martin , D. B. Matchar , M. E. Mussolino , S. D. Navaneethan , A. M. Perak , G. A. Roth , et al., Circulation 2021, 143, 254. 33501848 10.1161/CIR.0000000000000950 PMC13036842

[2] D. O. Kleindorfer , A. Towfighi , S. Chaturvedi , K. M. Cockroft , J. Gutierrez , D. Lombardi‐Hill , H. Kamel , W. N. Kernan , S. J. Kittner , E. C. Leira , O. Lennon , J. F. Meschia , T. N. Nguyen , P. M. Pollak , P. Santangeli , A. Z. Sharrief , S. C. Smith, Jr. , T. N. Turan , L. S. Williams , Stroke 2021, 52, 364. 10.1161/STR.0000000000000375 34024117

[3] G. Tsivgoulis , M. Saqqur , V. K. Sharma , A. Brunser , J. Eggers , R. Mikulik , A. H. Katsanos , T. N. Sergentanis , K. Vadikolias , F. Perren , M. Rubiera , R. Bavarsad Shahripour , H. T. Nguyen , P. Martínez‐Sánchez , A. Safouris , I. Heliopoulos , A. Shuaib , C. Derksen , K. Voumvourakis , T. Psaltopoulou , A. W. Alexandrov , A. V. Alexandrov , J. Stroke 2020, 22, 130. 32027798 10.5853/jos.2019.01648 PMC7005347

[4] Q. Z. Tuo , S. T. Zhang , P. Lei , Med. Res. Rev. 2022, 42, 259. 33957000 10.1002/med.21817

[5] C. Qin , L. Q. Zhou , X. T. Ma , Z. W. Hu , S. Yang , M. Chen , D. B. Bosco , L. J. Wu , D. S. Tian , Neurosci. Bull. 2019, 35, 921. 31062335 10.1007/s12264-019-00388-3 PMC6754485

[6] L. Jin , Z. Zhu , L. Hong , Z. Qian , F. Wang , Z. Mao , Bioact. Mater. 2023, 19, 38. 35415314 10.1016/j.bioactmat.2022.03.040 PMC8980441

[7] Y. Tang , J. Liu , Y. Wang , L. Yang , B. Han , Y. Zhang , Y. Bai , L. Shen , M. Li , T. Jiang , Q. Ye , X. Yu , R. Huang , Z. Zhang , Y. Xu , H. Yao , Autophagy 2021, 17, 2905. 33317392 10.1080/15548627.2020.1847799 PMC8525999

[8] W. Chen , Y. Zhang , X. Zhai , L. Xie , Y. Guo , C. Chen , Y. Li , F. Wang , Z. Zhu , L. Zheng , J. Wan , P. Li , J. Cereb. Blood Flow Metab. 2022, 42, 1579. 35491825 10.1177/0271678X221098841 PMC9441720

[9] W. Li , N. Shen , L. Kong , H. Huang , X. Wang , Y. Zhang , G. Wang , P. Xu , W. Hu , Stroke Vasc. Neurol. 2024, 9, 153. 37402504 10.1136/svn-2023-002320 PMC11103158

[10] Y. Wang , Z. Liu , L. Li , Z. Zhang , K. Zhang , M. Chu , Y. Liu , X. Mao , D. Wu , D. Xu , J. Zhao , J. Nanobiotechnol. 2024, 22, 291. 10.1186/s12951-024-02560-y PMC11129432 38802919

[11] A. S. Milliken , S. M. Nadtochiy , P. S. Brookes , Am. Heart Assoc. 2022, 11, 026135. 10.1161/JAHA.122.026135 PMC9333399 35766275

[12] V. Brinkmann , U. Reichard , C. Goosmann , B. Fauler , Y. Uhlemann , D. S. Weiss , Y. Weinrauch , A. Zychlinsky , Science 2004, 303, 1532. 15001782 10.1126/science.1092385

[13] H. R. Thiam , S. L. Wong , D. D. Wagner , C. M. Waterman , Annu. Rev. Cell Dev. Biol. 2020, 36, 191. 32663035 10.1146/annurev-cellbio-020520-111016 PMC8499668

[14] E. Ramos‐Martínez , L. Hernández‐González , I. Ramos‐Martínez , L. Pérez‐Campos Mayoral , G. I. López‐Cortés , E. Pérez‐Campos , G. Mayoral Andrade , M. T. Hernández‐Huerta , M. V. José , Front. Immunol. 2021, 12, 621311. 33717121 10.3389/fimmu.2021.621311 PMC7943724

[15] P. Michel‐Flutot , C. H. Bourcier , L. Emam , A. Gasser , S. Glatigny , S. Vinit , A. Mansart , Eur. J. Neurosci. 2023, 57, 692. 36537022 10.1111/ejn.15902

[16] C. Wang , Y. Wang , X. Shi , X. Tang , W. Cheng , X. Wang , Y. An , S. Li , H. Xu , Y. Li , W. Luan , X. Wang , Z. Chen , M. Liu , L. Yu , Front. Cell Neurosci. 2019, 13, 199. 31133815 10.3389/fncel.2019.00199 PMC6516055

[17] I. Agrawal , N. Sharma , S. Saxena , S. Arvind , D. Chakraborty , D. B. Chakraborty , D. Jha , S. Ghatak , S. Epari , T. Gupta , S. Jha , iScience 2021, 24, 101968. 33458617 10.1016/j.isci.2020.101968 PMC7797945

[18] D. Stojkov , P. Amini , K. Oberson , C. Sokollik , A. Duppenthaler , H. U. Simon , S. Yousefi , J. Cell Biol. 2017, 216, 4073. 29150539 10.1083/jcb.201611168 PMC5716265

[19] E. Neubert , D. Meyer , F. Rocca , G. Günay , A. Kwaczala‐Tessmann , J. Grandke , S. Senger‐Sander , C. Geisler , A. Egner , M. P. Schön , L. Erpenbeck , S. Kruss , Nat. Commun. 2018, 9, 3767. 30218080 10.1038/s41467-018-06263-5 PMC6138659

[20] K. D. Metzler , C. Goosmann , A. Lubojemska , A. Zychlinsky , V. Papayannopoulos , Cell Rep. 2014, 8, 883. 25066128 10.1016/j.celrep.2014.06.044 PMC4471680

[21] V. Papayannopoulos , K. D. Metzler , A. Hakkim , A. Zychlinsky , J. Cell Biol. 2010, 191, 677. 20974816 10.1083/jcb.201006052 PMC3003309

[22] I. Neeli , M. Radic , Front. Immunol. 2013, 4, 38. 23430963 10.3389/fimmu.2013.00038 PMC3576869

[23] I. Neeli , S. N. Khan , M. Radic , J. Immunol. 2008, 180, 1895. 18209087 10.4049/jimmunol.180.3.1895

[24] E. T. Chouchani , V. R. Pell , E. Gaude , D. Aksentijević , S. Y. Sundier , E. L. Robb , A. Logan , S. M. Nadtochiy , E. N. J. Ord , A. C. Smith , F. Eyassu , R. Shirley , C. H. Hu , A. J. Dare , A. M. James , S. Rogatti , R. C. Hartley , S. Eaton , A. S. H. Costa , P. S. Brookes , S. M. Davidson , M. R. Duchen , K. Saeb‐Parsy , M. J. Shattock , A. J. Robinson , L. M. Work , C. Frezza , T. Krieg , M. P. Murphy , Nature 2014, 515, 431. 25383517 10.1038/nature13909 PMC4255242

[25] W. Yu , D. Gao , W. Jin , S. Liu , S. Qi , Neurochem. Res. 2018, 43, 420. 29168092 10.1007/s11064-017-2437-z

[26] H. A. Prag , M. P. Murphy , T. Krieg , Basic Res. Cardiol. 2023, 118, 34. 37639068 10.1007/s00395-023-01002-4 PMC10462584

[27] V. Chavda , B. Lu , Antioxidants (Basel) 2023, 12, 895. 37107270 10.3390/antiox12040895 PMC10135819

[28] S. Nair , K. S. Sobotka , P. Joshi , P. Gressens , B. Fleiss , C. Thornton , C. Mallard , H. Hagberg , Glia 2019, 67, 1047. 30637805 10.1002/glia.23587

[29] E. A. Barbu , V. M. Dominical , L. Mendelsohn , S. L. Thein , Front Immunol 2020, 11,1335. 32765493 10.3389/fimmu.2020.01335 PMC7378400

[30] E. C. Britt , J. Lika , M. A. Giese , T. J. Schoen , G. L. Seim , Z. Huang , P. Y. Lee , A. Huttenlocher , J. Fan , Nat. Metab. 2022, 4, 389. 35347316 10.1038/s42255-022-00550-8 PMC8964420

[31] D. Liu , M. Xiao , J. Zhou , P. Wang , J. Peng , W. Mao , Y. Hu , Y. Liu , J. Yin , L. Ke , W. Li , Int. Immunopharmacol. 2023, 123, 110737. 37543012 10.1016/j.intimp.2023.110737

[32] M. Dambrova , C. J. Zuurbier , V. Borutaite , E. Liepinsh , M. Makrecka‐Kuka , Free Radic. Biol. Med. 2021, 165, 24. 33484825 10.1016/j.freeradbiomed.2021.01.036

[33] Z. Zhang , Z. Lu , C. Liu , J. Man , X. Li , K. Cui , H. Lu , J. Wang , Neuroreport 2021, 32, 1161. 34334775 10.1097/WNR.0000000000001704

[34] M. Carlström , L. R. Ribeiro , A. Carvalho , D. Guimaraes , A. Boeder , T. A. Schiffer , Redox Biol. 2024, 69, 102984. 38061207 10.1016/j.redox.2023.102984 PMC10749277

[35] X. Gao , G. Su , M. Chai , M. Shen , Z. Hu , W. Chen , J. Gao , R. Li , T. Ma , Y. An , Z. Zhang , Neurochem. Int. 2024, 172, 105656. 38081419 10.1016/j.neuint.2023.105656

[36] J. D. Adelson , G. E. Barreto , L. Xu , T. Kim , B. K. Brott , Y. B. Ouyang , T. Naserke , M. Djurisic , X. Xiong , C. J. Shatz , R. G. Giffard , Neuron 2012, 73, 1100. 22445338 10.1016/j.neuron.2012.01.020 PMC3314229

[37] K. Zheng , L. Lin , W. Jiang , L. Chen , X. Zhang , Q. Zhang , Y. Ren , J. Hao , J. Cereb. Blood Flow Metab. 2022, 42, 56. 34496660 10.1177/0271678X211026770 PMC8721774

[38] L. Kang , H. Yu , X. Yang , Y. Zhu , X. Bai , R. Wang , Y. Cao , H. Xu , H. Luo , L. Lu , M. J. Shi , Y. Tian , W. Fan , B. Q. Zhao , Nat. Commun. 2020, 11, 2488. 32427863 10.1038/s41467-020-16191-y PMC7237502

[39] M. De Wilde , L. Desender , C. Tersteeg , K. Vanhoorelbeke , S. F. De Meyer , Res. Pract. Thromb. Haemost. 2023, 7, 100028. 36852112 10.1016/j.rpth.2022.100028 PMC9958086

[40] G. Enzmann , C. Mysiorek , R. Gorina , Y. J. Cheng , S. Ghavampour , M. J. Hannocks , V. Prinz , U. Dirnagl , M. Endres , M. Prinz , R. Beschorner , P. N. Harter , M. Mittelbronn , B. Engelhardt , L. Sorokin , Acta Neuropathol. 2013, 125, 395. 23269317 10.1007/s00401-012-1076-3 PMC3578720

[41] V. Papayannopoulos , Nat. Rev. Immunol. 2018, 18, 134. 28990587 10.1038/nri.2017.105

[42] N. Vorobjeva , I. Galkin , O. Pletjushkina , S. Golyshev , R. Zinovkin , A. Prikhodko , V. Pinegin , I. Kondratenko , B. Pinegin , B. Chernyak , Biochim. Biophys. Acta, Mol. Basis Dis. 2020, 1866, 165664. 31926265 10.1016/j.bbadis.2020.165664

[43] Y. Wang , W. Wang , N. Wang , A. R. Tall , I. Tabas , Arterioscler., Thromb., Vasc. Biol. 2017, 37, 99. 10.1161/ATVBAHA.117.309580 PMC5535797 28596373

[44] N. Vorobjeva , A. Prikhodko , I. Galkin , O. Pletjushkina , R. Zinovkin , G. Sud'ina , B. Chernyak , B. Pinegin , Eur. J. Cell Biol. 2017, 96, 254. 28325500 10.1016/j.ejcb.2017.03.003

[45] K. A. Fortner , L. P. Blanco , I. Buskiewicz , N. Huang , P. C. Gibson , D. L. Cook , H. L. Pedersen , P. S. T. Yuen , M. P. Murphy , A. Perl , M. J. Kaplan , R. C. Budd , Lupus Sci. Med. 2020, 7, 000387. 10.1136/lupus-2020-000387 PMC7199895 32343673

[46] S. Kröller‐Schön , S. Steven , S. Kossmann , A. Scholz , S. Daub , M. Oelze , N. Xia , M. Hausding , Y. Mikhed , E. Zinssius , M. Mader , P. Stamm , N. Treiber , K. Scharffetter‐Kochanek , H. Li , E. Schulz , P. Wenzel , T. Münzel , A. Daiber , Antioxid. Redox Signaling 2014, 20, 247. 10.1089/ars.2012.4953 PMC3887465 23845067

[47] A. K. Doughan , D. G. Harrison , S. I. Dikalov , Circ. Res. 2008, 102, 488. 18096818 10.1161/CIRCRESAHA.107.162800

[48] A. Mottahedin , H. A. Prag , A. Dannhorn , R. Mair , C. Schmidt , M. Yang , A. Sorby‐Adams , J. J. Lee , N. Burger , D. Kulaveerasingam , M. M. Huang , S. Pluchino , L. Peruzzotti‐Jametti , R. Goodwin , C. Frezza , M. P. Murphy , T. Krieg , Redox Biol. 2023, 59, 102600. 36630820 10.1016/j.redox.2023.102600 PMC9841348

[49] P. W. Hochachka , R. H. Dressendorfer , Eur. J. Appl. Physiol. Occup. Physiol. 1976, 35, 235. 976251 10.1007/BF00423282

[50] J. Kamarauskaite , R. Baniene , D. Trumbeckas , A. Strazdauskas , S. Trumbeckaite , Biomed Res. Int. 2020, 2020, 8855585. 33102598 10.1155/2020/8855585 PMC7578729

[51] C. Hohl , R. Oestreich , P. Rösen , R. Wiesner , M. Grieshaber , Arch. Biochem. Biophys. 1987, 259, 527. 3426243 10.1016/0003-9861(87)90519-4

[52] J. Zhang , Y. T. Wang , J. H. Miller , M. M. Day , J. C. Munger , P. S. Brookes , Cell Rep. 2018, 23, 2617. 29847793 10.1016/j.celrep.2018.04.104 PMC6002783

[53] E. T. Chouchani , V. R. Pell , A. M. James , L. M. Work , K. Saeb‐Parsy , C. Frezza , T. Krieg , M. P. Murphy , Cell Metab. 2016, 23, 254. 26777689 10.1016/j.cmet.2015.12.009

[54] T. E. Beach , H. A. Prag , L. Pala , A. Logan , M. M. Huang , A. V. Gruszczyk , J. L. Martin , K. Mahbubani , M. O. Hamed , S. A. Hosgood , M. L. Nicholson , A. M. James , R. C. Hartley , M. P. Murphy , K. Saeb‐Parsy , Redox Biol. 2020, 36, 101640. 32863205 10.1016/j.redox.2020.101640 PMC7372157

[55] H. A. Prag , D. Aksentijevic , A. Dannhorn , A. V. Giles , J. F. Mulvey , O. Sauchanka , L. Du , G. Bates , J. Reinhold , D. Kula‐Alwar , Z. Xu , L. Pellerin , R. J. A. Goodwin , M. P. Murphy , T. Krieg , Circ. Res. 2022, 131, 528. 35959683 10.1161/CIRCRESAHA.121.320717 PMC9426742

[56] F. J. B. Miranda , B. C. Rocha , M. C. A. Pereira , L. M. N. Pereira , E. H. M. de Souza , A. P. Marino , P. A. C. Costa , D. V. Vasconcelos‐Santos , L. R. V. Antonelli , R. T. Gazzinelli , mBio 2021, 12, 0130721. 10.1128/mBio.01307-21 PMC8546543 34607465

[57] S. Sharma , T. M. Hofbauer , A. S. Ondracek , S. Chausheva , A. Alimohammadi , T. Artner , A. Panzenboeck , J. Rinderer , I. Shafran , A. Mangold , R. Winker , E. Wohlschläger‐Krenn , B. Moser , S. Taghavi , W. Klepetko , K. T. Preissner , I. M. Lang , Blood 2021, 137, 1104. 33512471 10.1182/blood.2020005861

[58] K. Okubo , M. Kurosawa , M. Kamiya , Y. Urano , A. Suzuki , K. Yamamoto , K. Hase , K. Homma , J. Sasaki , H. Miyauchi , T. Hoshino , M. Hayashi , T. N. Mayadas , J. Hirahashi , Nat. Med. 2018, 24, 232. 29309057 10.1038/nm.4462

[59] P. Burkard , C. Schonhart , T. Vögtle , D. Köhler , L. Tang , D. Johnson , K. Hemmen , K. G. Heinze , A. Zarbock , H. M. Hermanns , P. Rosenberger , B. Nieswandt , Blood 2023, 142, 1463. 37441848 10.1182/blood.2023019940

[60] Y. Lu , Y. Huang , J. Li , J. Huang , L. Zhang , J. Feng , J. Li , Q. Xia , Q. Zhao , L. Huang , S. Jiang , S. Su , Nat. Cell Biol. 2021, 23, 1060. 34616019 10.1038/s41556-021-00762-2

[61] Y. Döring , O. Soehnlein , C. Weber , Circ. Res. 2017, 120, 736. 28209798 10.1161/CIRCRESAHA.116.309692

[62] S. Yang , S. Wang , L. Chen , Z. Wang , J. Chen , Q. Ni , X. Guo , L. Zhang , G. Xue , Int. J. Biol. Sci. 2023, 19, 347. 36594092 10.7150/ijbs.78046 PMC9760440

[63] A. Hervera , F. De Virgiliis , I. Palmisano , L. Zhou , E. Tantardini , G. Kong , T. Hutson , M. C. Danzi , R. B. Perry , C. X. C. Santos , A. N. Kapustin , R. A. Fleck , J. A. Del Río , T. Carroll , V. Lemmon , J. L. Bixby , A. M. Shah , M. Fainzilber , S. Di Giovanni , Nat. Cell Biol. 2018, 20, 307. 29434374 10.1038/s41556-018-0039-x

[64] C. Lood , L. P. Blanco , M. M. Purmalek , C. Carmona‐Rivera , S. S. De Ravin , C. K. Smith , H. L. Malech , J. A. Ledbetter , K. B. Elkon , M. J. Kaplan , Nat. Med. 2016, 22, 146. 26779811 10.1038/nm.4027 PMC4742415

[65] S. Sugiyama , K. Kugiyama , M. Aikawa , S. Nakamura , H. Ogawa , P. Libby , Arterioscler., Thromb., Vasc. Biol. 2004, 24, 1309. 15142860 10.1161/01.ATV.0000131784.50633.4f

[66] L. F. Pereira , F. M. Marco , R. Boimorto , A. Caturla , A. Bustos , E. G. De la Concha , J. L. Subiza , Clin. Exp. Immunol. 1994, 97, 175. 8050163 10.1111/j.1365-2249.1994.tb06064.x PMC1534698

[67] S. Wu , J. Yang , G. Sun , J. Hu , Q. Zhang , J. Cai , D. Yuan , H. Li , Z. Hei , W. Yao , Br. J. Pharmacol. 2021, 178, 3783. 33959955 10.1111/bph.15518

[68] J. Singh , L. Zlatar , M. Muñoz‐Becerra , G. Lochnit , I. Herrmann , F. Pfister , C. Janko , J. Knopf , M. Leppkes , J. Schoen , L. E. Muñoz , G. Schett , M. Herrmann , C. Schauer , A. Mahajan , Cell Commun. Signaling 2024, 22, 435. 10.1186/s12964-024-01785-6 PMC11384698 39252008

[69] H. Li , Y. Li , C. Song , Y. Hu , M. Dai , B. Liu , P. Pan , J. Inflammation Res. 2021, 14, 4839. 10.2147/JIR.S321513 PMC8473117 34588792

[70] A. Stachowicz , R. Pandey , N. Sundararaman , V. Venkatraman , J. E. Van Eyk , J. Fert‐Bober , J. Inflammation (Lond) 2022, 19, 20. 10.1186/s12950-022-00317-8 PMC9675280 36401289

[71] P. Conde , M. Rodriguez , W. van der Touw , A. Jimenez , M. Burns , J. Miller , M. Brahmachary , H. M. Chen , P. Boros , F. Rausell‐Palamos , T. J. Yun , P. Riquelme , A. Rastrojo , B. Aguado , J. Stein‐Streilein , M. Tanaka , L. Zhou , J. Zhang , T. L. Lowary , F. Ginhoux , C. G. Park , C. Cheong , J. Brody , S. J. Turley , S. A. Lira , V. Bronte , S. Gordon , P. S. Heeger , M. Merad , J. Hutchinson , Immunity 2015, 42, 1143. 26070485 10.1016/j.immuni.2015.05.009 PMC4690204

[72] X. Wu , Y. Guo , H. Zeng , G. Chen , J. Clin. Med. 2022, 11, 4349. 35955969 10.3390/jcm11154349 PMC9369252

[73] Q. Z. Tuo , P. Lei , K. A. Jackman , X. L. Li , H. Xiong , X. L. Li , Z. Y. Liuyang , L. Roisman , S. T. Zhang , S. Ayton , Q. Wang , P. J. Crouch , K. Ganio , X. C. Wang , L. Pei , P. A. Adlard , Y. M. Lu , R. Cappai , J. Z. Wang , R. Liu , A. I. Bush , Mol. Psychiatry 2017, 22, 1520. 28886009 10.1038/mp.2017.171

[74] T. Chiang , R. O. Messing , W.‐H. Chou , J. Visualized Exp. 2011, 13, 2761. 10.3791/2761 PMC3197421 21372780

[75] Y.‐H. Shi , X.‐L. Zhang , P.‐J. Ying , Z.‐Q. Wu , L.‐L. Lin , W. Chen , G.‐Q. Zheng , W.‐Z. Zhu , Front. Pharmacol. 2021, 12, 639898. 33841157 10.3389/fphar.2021.639898 PMC8033022

[76] W. B. Dunn , D. Broadhurst , P. Begley , E. Zelena , S. Francis‐McIntyre , N. Anderson , M. Brown , J. D. Knowles , A. Halsall , J. N. Haselden , A. W. Nicholls , I. D. Wilson , D. B. Kell , R. Goodacre , Nat. Protoc. 2011, 6, 1060. 21720319 10.1038/nprot.2011.335

[77] Y. Zhang , D. Zhu , T. Li , X. Wang , L. Zhao , X. Yang , M. Dang , Y. Li , Y. Wu , Z. Lu , J. Lu , Y. Jian , H. Wang , L. Zhang , X. Lu , Z. Shen , H. Fan , W. Cai , G. Zhang , Biomed. Pharmacother. 2022, 155, 113641. 36088854 10.1016/j.biopha.2022.113641

[78] T. Li , L. Zhao , Y. Li , M. Dang , J. Lu , Z. Lu , Q. Huang , Y. Yang , Y. Feng , X. Wang , Y. Jian , H. Wang , Y. Guo , L. Zhang , Y. Jiang , S. Fan , S. Wu , H. Fan , F. Kuang , G. Zhang , Cell Death Dis. 2023, 14, 634. 37752100 10.1038/s41419-023-06135-x PMC10522625

[79] H. A. Prag , L. Pala , D. Kula‐Alwar , J. F. Mulvey , D. Luping , T. E. Beach , L. M. Booty , A. R. Hall , A. Logan , V. Sauchanka , S. T. Caldwell , E. L. Robb , A. M. James , Z. Xu , K. Saeb‐Parsy , R. C. Hartley , M. P. Murphy , T. Krieg , Cardiovasc. Drugs Ther. 2022, 36, 1. 32648168 10.1007/s10557-020-07033-6 PMC8770414

[80] Y. Yang , S. Kimura‐Ohba , J. Thompson , G. A. Rosenberg , Transl. Stroke Res. 2016, 7, 407. 27498679 10.1007/s12975-016-0486-2 PMC5016244

[81] C. Fei , D. M. E. Lillico , B. Hall , A. M. Rieger , J. L. Stafford , Cytometry A 2017, 91, 372. 28081295 10.1002/cyto.a.23050

[82] T. A. More , B. Dalal , R. Devendra , P. Warang , A. Shankarkumar , P. Kedar , Cytometry B Clin. Cytom. 2020, 98, 238. 31750618 10.1002/cyto.b.21857

[83] J. H. Garcia , S. Wagner , K. F. Liu , X. J. Hu , Stroke 1995, 26, 627. 7709410 10.1161/01.str.26.4.627

