# Supplementary material for: Succinate Dehydrogenase Subunit A (SDHA) Mediated Microglia Extracellular Traps Formation Participating in Cerebral Ischemic Reperfusion Injury
Source: Adv Sci (Weinh). 2025 Aug 20;12(35):e11873. doi: 10.1002/advs.202411873 (PMC12463099; doi:10.1002/advs.202411873)
Supplement: Supplementary file 1 — Supporting Information [file ADVS-12-e11873-s002.docx]

**Succinate dehydrogenase A subunit (SDHA) mediated microglia extracellular traps formation participating in cerebral ischemic reperfusion injury**

Lili Zhao^1, #^; Tao Li^1, #^; Meijuan Dang^1, #^; Ye Li ^1^; Jialiang Lu ^1^; Ziwei Lu ^1^; Zhiyang Chen^2^; Qiao Huang^2^; Yujie Chen^2^; Yang Yang^1^; Yuxuan Feng^1^; Xiaoya Wang^1^; Yating Jian^1^; Heying Wang^1^; Yingying Guo^3^; Lei Zhang^1^; Yu Jiang^1^; Songhua Fan^1^; Shengxi Wu^2^; Hong Fan ^1,^ *; Fang Kuang^2,^ *; Guilian Zhang ^1,^ *

^1^ Department of Neurology, the Second Affiliated Hospital of Xi'an Jiaotong University, Xi'an, 710004, Shaanxi, China

^2^ Department of Neurobiology, School of Basic Medicine, Fourth Military Medical University, 710032, Shaanxi, China.

^3^ Department of Pediatrics, the Second Affiliated Hospital of Xi'an Jiaotong University, Xi'an, 710004, Shaanxi, China

*Corresponding authors:

Hong Fan: fanhong_2005@126.com

Fang Kuang: kuangf@fmmu.edu.cn

Guilian Zhang: [zhgl_2006@xjtu.edu.cn](mailto:zhgl_2006@xjtu.edu.cn)

^#^ These authors have contributed equally to this work and share first authorship.

**Table S1** RT-PCR primer sequences

| Gene | Primer（5'-3'） |
| --- | --- |
| *SDHA*- Forward | TGCTCCTTTGGGAACCACAGCT |
| *SDHA*- Reverse | GAGATACGCACCTGTTGCCAAG |
| *SDHB*- Forward | TGCGGACCTATGGTGTTGGATG |
| *SDHB*- Reverse | CCAGAGTATTGCCTCCGTTGATG |
| *SDHC*- Forward | TGCTCCTTTGGGAACCACAGCT |
| *SDHC*- Reverse | GCAAACGGACAGTGCCATAGGA |
| *SDHD*- Forward | TGGTCAGACCCGCTTATGTG |
| *SDHD*- Reverse | GGTCCAGTGGAGAGATGCAG |
| *β-actin*-Forward | CATTGCTGACAGGATGCAGAAGG |
| *β-actin*-Reverse | TGCTGGAAGGTGGACAGTGAGG |

**Table S2** The sequences of shRNA

| Marker | Gene | Gene ID | TargetSeq |
| --- | --- | --- | --- |
| Y38637 | SDHA | NM_023281.1 | GCATCAGCTAAAGTTTCAGAT |
| GL427NC2 | NC2 | NA | CCTAAGGTTAAGTCGCCCTCG |


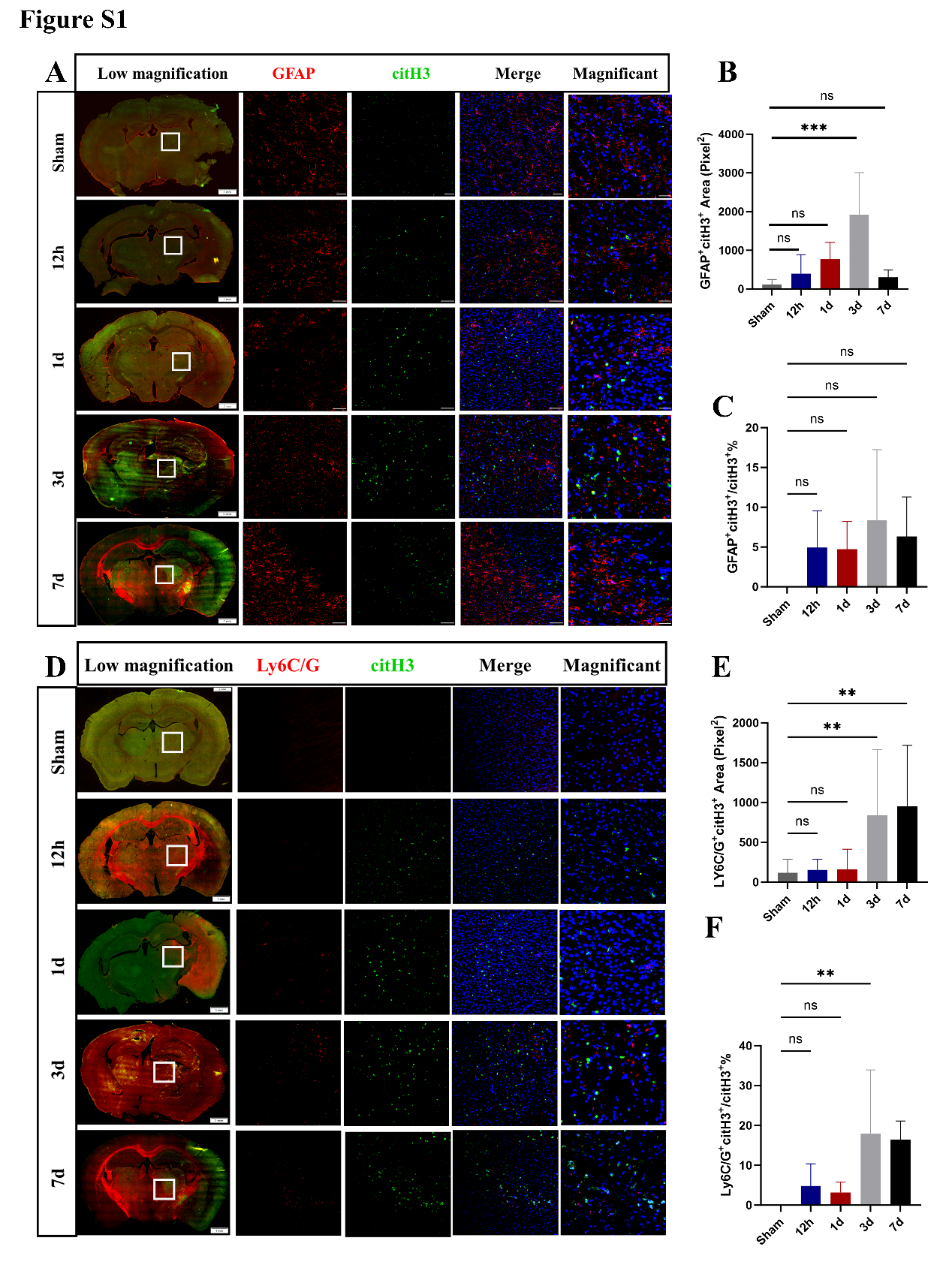


**Figure S1** **The expression of citH3 in astrocytes and macrophages/monocytes at different time points after cerebral I/R**. (**A**) Immunofluorescence staining for GFAP (red), with citH3 (green) at 12h and 1, 3, 7 days post cerebral I/R in sham and I/R groups in peri-infarction area. Scale bar = 100 μm. (**B, C**) Quantification of GFAP/citH3 double positive area and percentage of GFAP^+^citH3^+^ at different time points (n = 9–16 images from four animals/group, one-way ANOVA followed by multiple comparisons). (**D**) Immunofluorescence staining for Ly6C/G (red), with citH3 (green) at 12h and 1, 3, 7 days post cerebral I/R in sham and I/R groups in peri-infarction area. Scale bar = 100 μm. (**E, F**) Quantification of Ly6C/G/citH3 double positive area and percentage of GFAP^+^citH3^+^ at different time points (n = 9–16 images from four animals/group, one-way ANOVA followed by multiple comparisons). All data are presented as mean ± SD, ***P*<0.01.


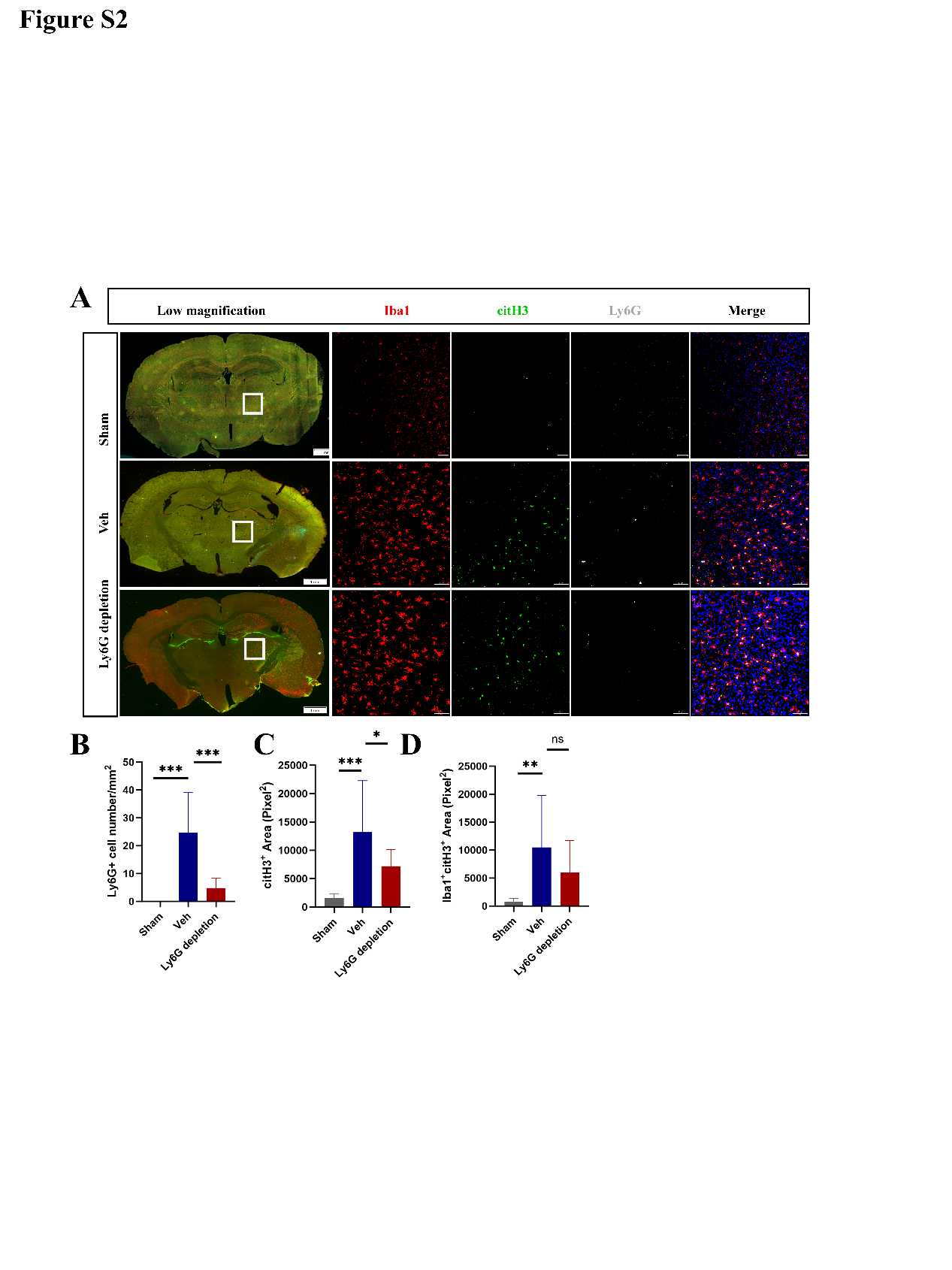


**Figure S2** **The depletion of neutrophils has no impact on MiETs content following cerebral I/R at 24 h**. (**A**) Immunofluorescence staining for Iba1 (red), Ly6G (Gray) with citH3 (green) at 24 hours post cerebral I/R in sham and I/R groups in peri-infarction area. Scale bar = 100 μm. (**B-D**) Quantification of Ly6G^+^ cell number, citH3^+^ area, Iba1/citH3 double positive area in three groups (n = 9–12 images from four animals/group, one-way ANOVA followed by multiple comparisons). All data are presented as mean ± SD, ***P*<0.01.


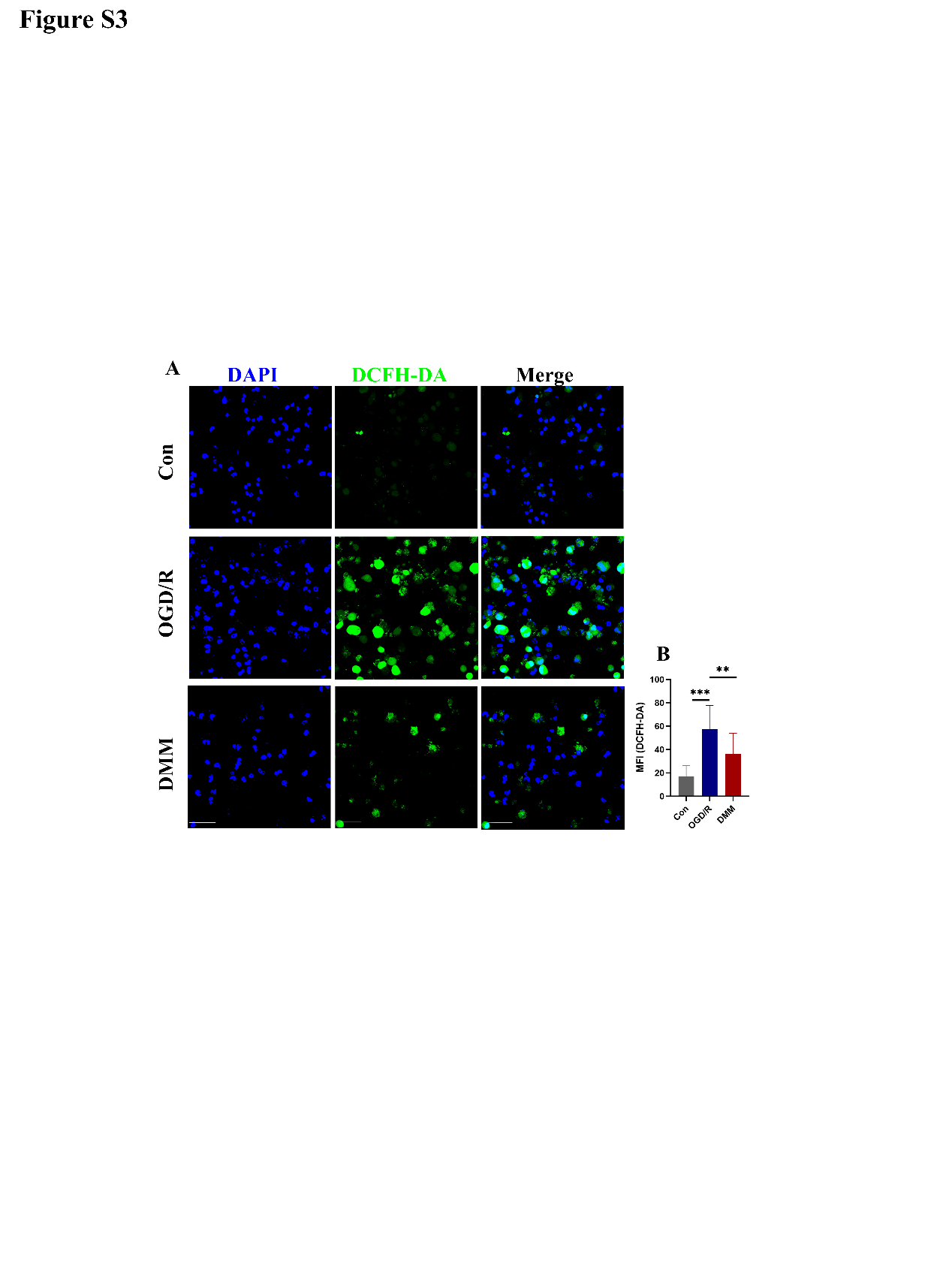


**Figure S3** **OGD/R induced increased cytosolic ROS in microglia and DMM decreased cytosolic level of ROS**. (**A**): Representative images of DCFH-DA-stained microglia treated with OGD/R and quantitative analysis of mean immunofluorescence intensities (MFI) of DCFH-DA-positive staining in each group. (**B**): Representative images of DCFH-DA-stained microglia treated with OGD/R and DMM and statistical analysis of MFI in three groups. All experiments were performed at least three independent times. All data are presented as mean ± SD, ***P*<0.01, ****P*<0.001.


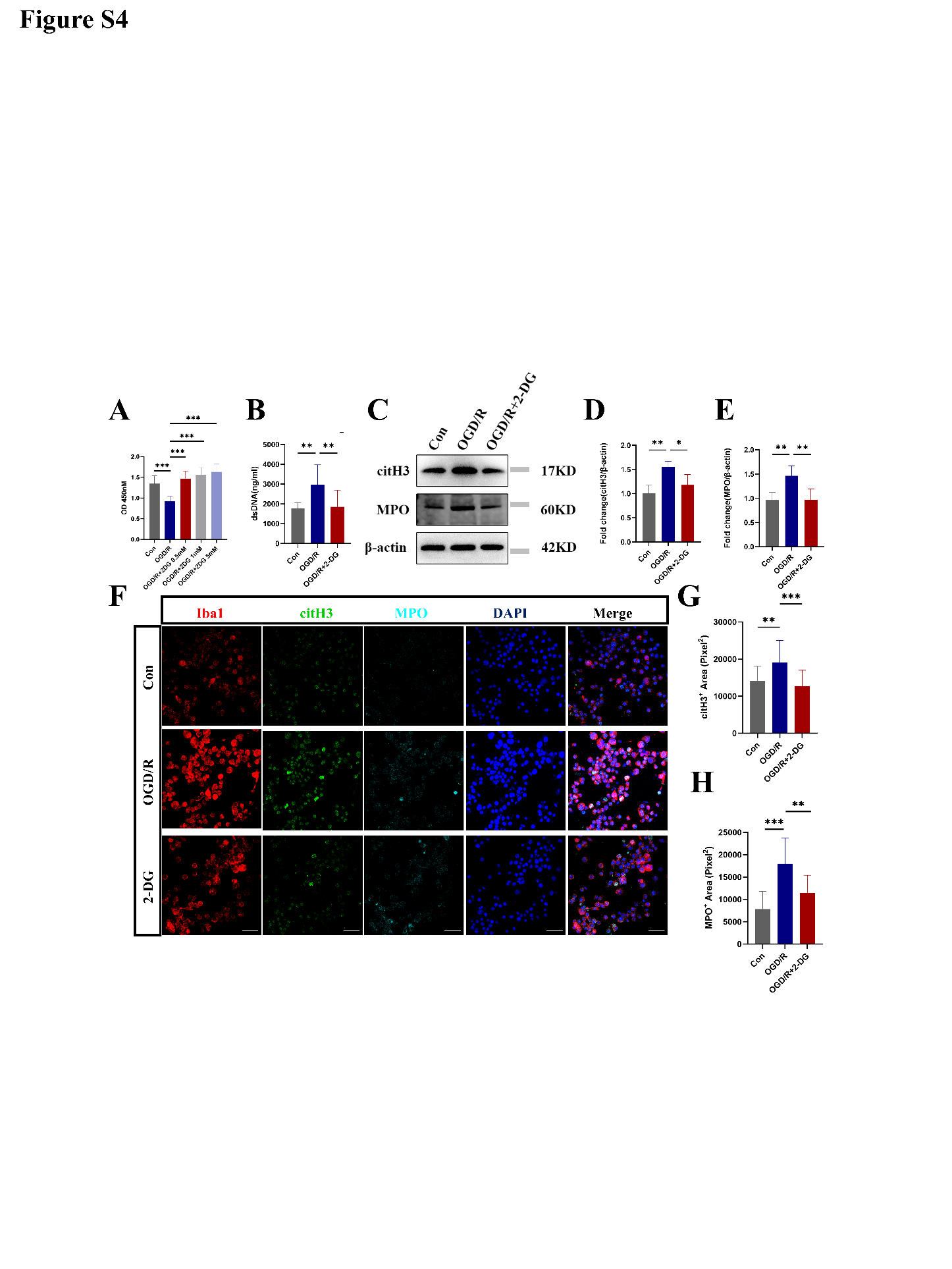


**Figure S4 Glycolysis inhibition by 2-DG attenuates MiETs production. (A)** Cell viability of microglia was detected by CCK-8 assay following incubation with 0.5, 1, 5 mM 2-DG. (B) Effect of 2-DG on dsDNA content in cell culture supernatant. (**C-E**) The representative picture of immunoblots and quantification analysis for citH3 and MPO in microglia after 2-DG treatment. (**F-H**) Representative images of Iba1/citH3/MPO triple staining and quantification of citH3/MPO area. Scale bar = 50 μm. All experiments of microglia were performed at least three independent times. All data are presented as mean ± SD, **P* < 0.05, ***P* < 0.01, ****P* < 0.001.


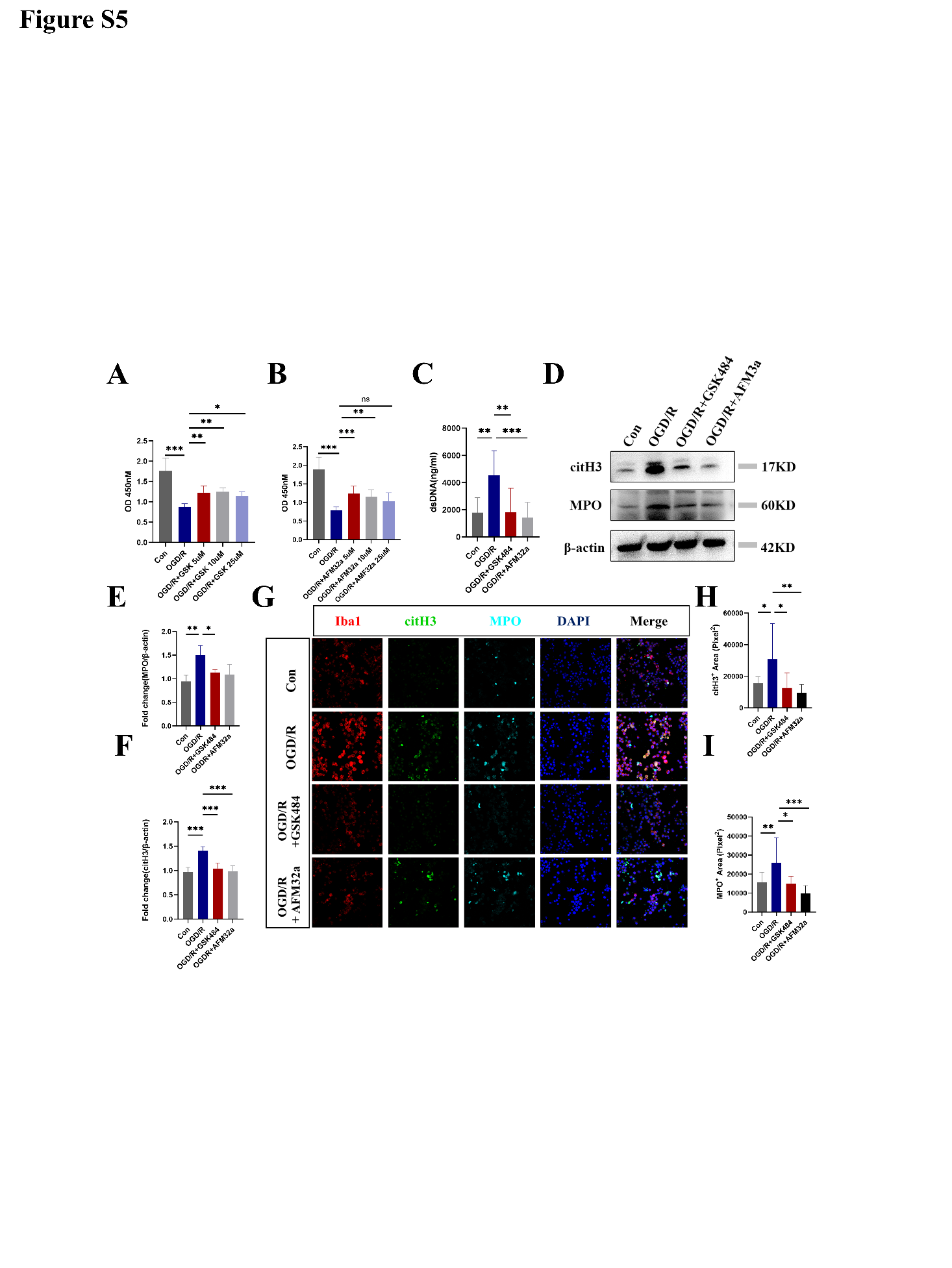


**Figure S5 Targeting PAD4 or PAD2 enzymatic activity reduces MiETosis.** (**A, B**) Cell viability of microglia was detected by CCK-8 assay following incubation with 5, 10, 25 uM GSK484 or 5, 10, 25 uM AFM32a. (**C**) Effect of GSK484 or AFM32a on dsDNA content in cell culture supernatant. (D-F) The representative picture of immunoblots and quantification analysis for citH3 and MPO in microglia after GSK484 or AFM32a treatment. (**G-I**) Representative images of Iba1/citH3/MPO triple staining and quantification of citH3/MPO area. Scale bar = 50 μm. All experiments of microglia were performed at least three independent times. All data are presented as mean ± SD, **P* < 0.05, ***P* < 0.01, ****P* < 0.001.


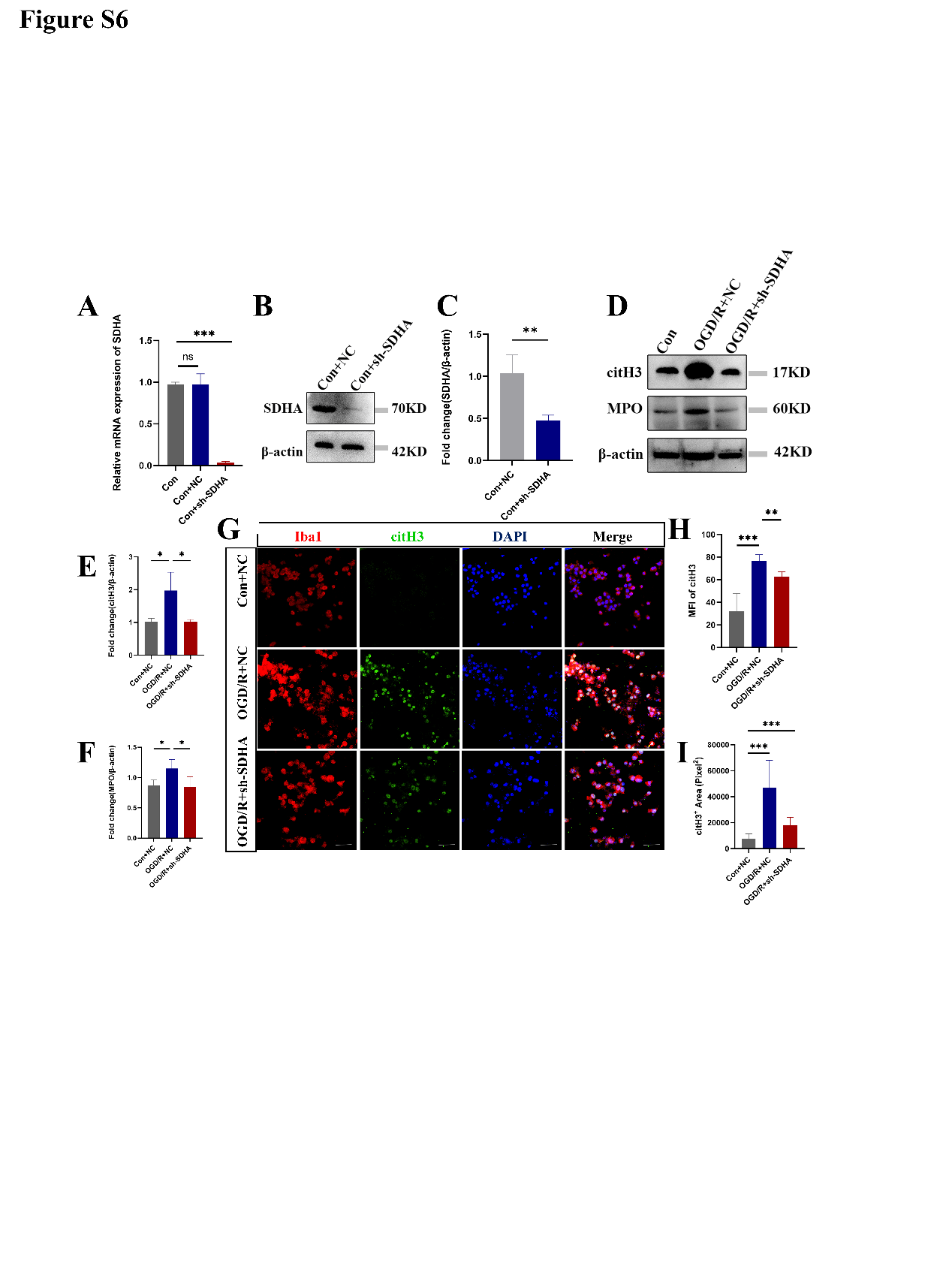


**Figure S6** **SDHA deficiency attenuates the production of MiETs induced by (OGD/R).** (**A**) mRNA expression of SDHA in micrglia treated with medium, infected with Lenti-vector, or Lenti-sh-SDHA. (**B, C**) Representative immunoblot and statistical analysis of SDHA in microglia treated medium, Lenti-vector, or Lenti-sh-SDHA. (**D-F**) The representative picture of immunoblots and quantification analysis for citH3 and MPO. (**G-I**) Representative images of Iba1/citH3 double staining and quantification MFI and area of citH3. Scale bar = 50 μm. All experiments of microglia were performed at least three independent times. All data are presented as mean ± SD, *P < 0.05, **P < 0.01, ***P < 0.001.


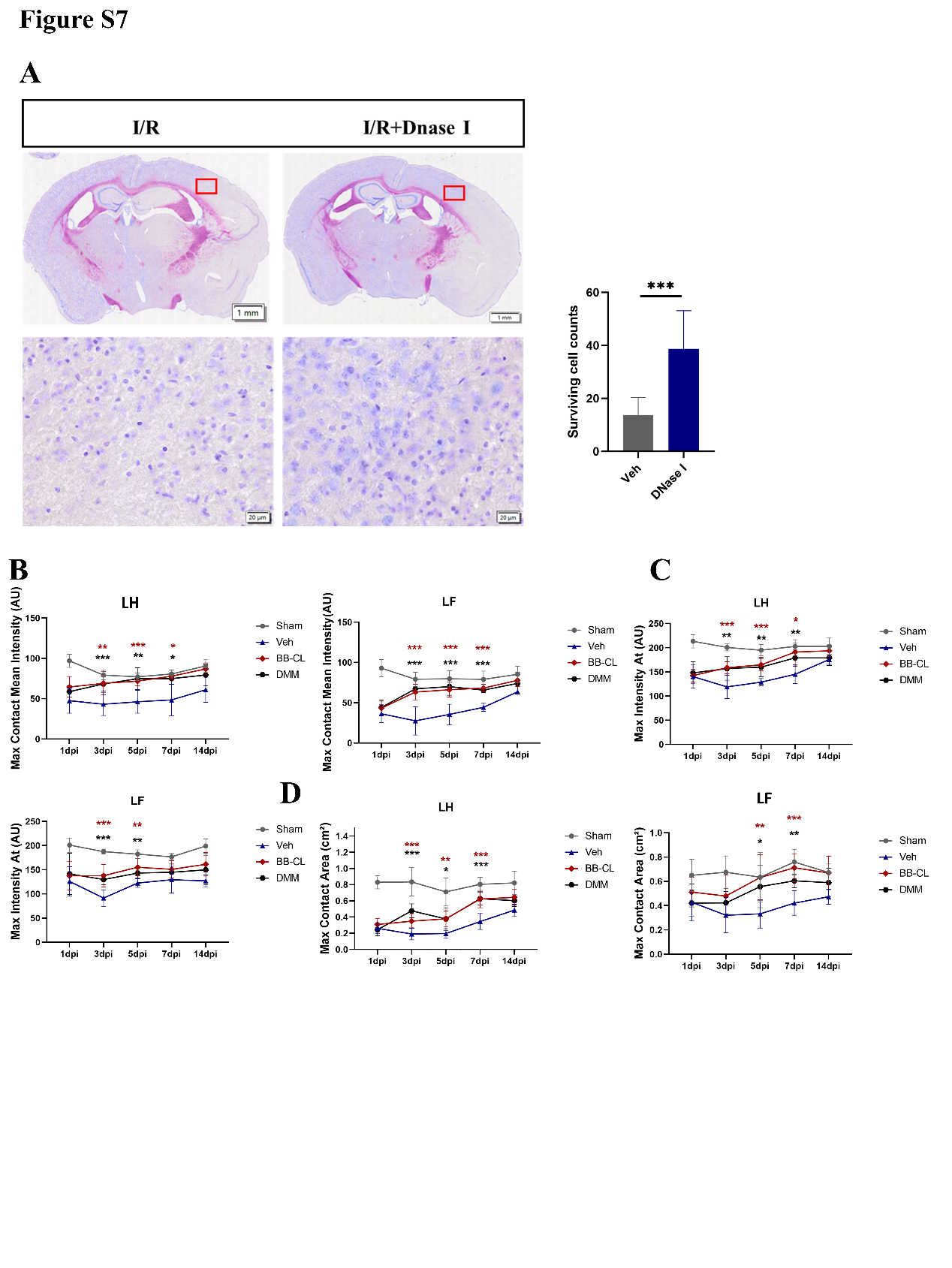


**Figure S7** **Inhibition or clearance of MiETs promoted motor functional recovery after cerebral I/R in mice**. (A) Representative images of Nissl staining and quantification of surviving cells among Veh, and DNase I groups. Scale bar = 1 mm in the upper panel and scale bar = 20 μm in the lower panel (n = 18 images from four animals/group, one-way ANOVA followed by multiple comparisons). Gait analysis showed DMM and BB-Cl significantly increased max contact mean intensity (**B**), max intensity at (**C**), max contact area (**D**) of the left forepaw and left hindpaw compared with the Veh group. n = 10-13/group at beginning, two-way ANOVA (Bonferroni’s multiple comparison test). LF: left forepaw, LH: left hindpaw. All data are presented as mean ± SD, **P*<0.05, ***P*<0.01, ****P*<0.001.
